# Reduction of aberrant NF-κB signalling ameliorates Rett syndrome phenotypes in *Mecp2*-null mice

**DOI:** 10.1038/ncomms10520

**Published:** 2016-01-29

**Authors:** Noriyuki Kishi, Jessica L. MacDonald, Julia Ye, Bradley J. Molyneaux, Eiman Azim, Jeffrey D. Macklis

**Affiliations:** 1 Department of Stem Cell and Regenerative Biology, Center for Brain Science, and Harvard Stem Cell Institute, Harvard University, Cambridge, Massachusetts 02138, USA

## Abstract

Mutations in the transcriptional regulator *Mecp2* cause the severe X-linked neurodevelopmental disorder Rett syndrome (RTT). In this study, we investigate genes that function downstream of MeCP2 in cerebral cortex circuitry, and identify upregulation of *Irak1*, a central component of the NF-κB pathway. We show that overexpression of *Irak1* mimics the reduced dendritic complexity of *Mecp2*-null cortical callosal projection neurons (CPN), and that NF-κB signalling is upregulated in the cortex with *Mecp2* loss-of-function. Strikingly, we find that genetically reducing NF-κB signalling in *Mecp2*-null mice not only ameliorates CPN dendritic complexity but also substantially extends their normally shortened lifespan, indicating broader roles for NF-κB signalling in RTT pathogenesis. These results provide new insight into both the fundamental neurobiology of RTT, and potential therapeutic strategies via NF-κB pathway modulation.

## Abstract (web summary)

Rett syndrome is a neurodevelopmental disorder caused by mutations in *Mecp2*. Here the authors show that *Mecp2* loss-of-function leads to upregulation of the NF-κB pathway, and that reducing NF-κB signalling ameliorates phenotypes of *Mecp2*-null mice, thus offering a potential therapeutic strategy.

## Graphical abstract

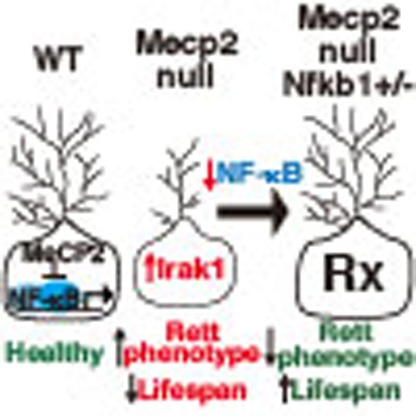

Rett syndrome (RTT) is an X-linked neurodevelopmental disorder presenting almost exclusively in girls, with a prevalence rate of one in 10,000–15,000 (ref. 1). After Down's syndrome, RTT is considered the second most common genetic cause of cognitive disability in girls. Children with RTT develop relatively normally for 6–18 months, after which they undergo a period of rapid regression, with loss of purposeful hand use, deceleration of head growth and onset of repetitive, autistic behaviours. Mutations of the *MECP2* gene on the X chromosome are found in over 95% of cases of classic RTT (refs 1, 2), and MECP2 has been implicated in several other neurodevelopmental disorders, including autism, childhood schizophrenia and X-linked cognitive disability^3^. Although mouse genetic studies clearly reveal that abnormal neurodevelopmental phenotypes of *Mecp2* mutant mice are largely attributable to lack of MeCP2 function in the central nervous system (CNS)^4^,^5^, there is little neuropathological understanding of the molecular causes of CNS abnormalities of *Mecp2*-null mice, or of children with RTT.

MeCP2 has been typically referred to as a transcriptional repressor that selectively binds to methyl-CpG dinucleotides in the mammalian genome and mediates transcriptional repression through interaction with co-factors; however, more recent work shows that MeCP2 can also function as a transcriptional activator^6^, and regulate global neuronal chromatin structure in response to neuronal activity^7^,^8^. Further, recent evidence clearly indicates that molecular pathways regulated by MeCP2 are tissue- and cell-type specific^9^,^10^,^11^,^12^, and that disruption of MeCP2 function in defined CNS circuits results in distinct RTT phenotypes^13^,^14^,^15^,^16^. Therefore, it is critical to identify MeCP2 target genes and pathways in distinct neuronal populations in order to understand the underlying molecular and cellular pathogenesis of RTT, and to design effective therapeutic strategies.

We have focused our investigation of molecular pathways regulated by MeCP2 on neocortical inter-hemispheric callosal projection neurons (CPN). CPN, projection neurons primarily located in cortical layers 2/3 (∼80%) and 5 (∼20%), connect homotopic regions of the two cerebral hemispheres via the corpus callosum. CPN are critically involved in bilateral associative integration of cortical information, and abnormal CPN development and dysfunction are implicated in autism^17^,^18^. Our previous results identified that layer 2/3 CPN increasingly express MeCP2 as they mature, and that loss of MeCP2 function reduces their dendritic complexity in a largely cell-autonomous manner^19^,^20^. Reduced dendritic complexity of neocortical layer 2/3 projection neurons (predominantly the inter-hemispheric CPN) has also been observed in post-mortem brains of RTT patients^21^,^22^, with synaptic circuit abnormalities identified in this population in mouse^23^. Thus, it seems likely that the morphological abnormalities observed in this neuronal population might underlie at least some aspects of the cognitive, integrative symptoms observed in RTT, as well as more broadly in autism. Together, these motivate and provide foundation for investigation and identification of gene targets of MeCP2 regulation in purified mouse CPN.

Focusing on CPN, we identify *Irak1*, a signalling kinase and scaffold protein within the NF-κB pathway, as one of the genes significantly upregulated by *Mecp2*-null CPN. We confirm that *Irak1* is upregulated following *Mecp2* loss-of-function, and that overexpression of *Irak1* recapitulates the reduced dendritic complexity phenotype of *Mecp2*-null CPN, both *in vitro* and *in vivo*. We establish that NF-κB pathway signalling is upregulated with loss of *Mecp2* function or *Irak1* overexpression in cortical neurons. Importantly, we identify that genetic attenuation of the aberrant NF-κB signalling in *Mecp2*-null (*Mecp2−/*y) mice not only ameliorates the CPN dendritic complexity phenotype, it improves health, and thus significantly extends (∼50%) the usually shortened lifespan of *Mecp2*-null mice. These results indicate that abnormal activation of NF-κB signalling, via loss of *Mecp2* modulation of *Irak1* expression, is centrally involved in the pathogenesis of *Mecp2*-null mice and, therefore, likely RTT. These results provide new insight into both the fundamental neurobiology of RTT, and potential therapeutic strategies via NF-κB pathway modulation.

## Results

### Identification of dysregulated genes in Mecp2-null CPN

To identify potential target genes regulated directly or indirectly by MeCP2, we performed comparative gene expression analysis on *Mecp2*−/y (*Mecp2*-null) and wild-type CPN purified via fluorescence-activated cell sorting (FACS; Supplementary Fig. 1), using an approach we previously developed^24^,^25^. This approach of interrogating purified neuronal subpopulations reveals more extensive transcriptional disruptions in *Mecp2*-null brains than earlier studies performed on heterogeneous brain tissue homogenates^12^, because of the different target genes of MeCP2 in distinct cell types. We identified 18 overexpressed and 19 underexpressed candidates that are most severely dysregulated at very high levels of statistical stringency (>2 or <−2-fold dysregulation, *P*<0.001) by *Mecp2*-null CPN (Supplementary Tables 1 and 2), and an additional 38 overexpressed and 46 underexpressed genes in *Mecp2*-null CPN using a lower stringency of >1.5 or <−1.5-fold dysregulation, *P*<0.005 (Supplementary Tables 3 and 4). Consistent with accumulating evidence that MeCP2 deficiency causes abnormal neuronal development and maturation, these dysregulated candidate downstream targets include genes such as *Kif1b*, *Mcf2* and *Gsn*, which are already known to be involved in dendritic, cytoskeletal and synaptic formation, maturation and maintenance (Supplementary Table 5).

From this newly identified set of genes regulated by MeCP2 in CPN, we chose interleukin-1 receptor associated kinase 1 (*Irak1*) to further validate and functionally investigate in depth. *Irak1* is a central component of the NF-κB pathway, which regulates multiple aspects of neuronal process development, including dendritic complexity and synaptic plasticity^26^,^27^. NF-κB signalling has been extensively investigated in other systems, such as the immune system, and a number of its inhibitors have been identified and are already in use clinically. Therefore, we reasoned that these investigations might have the potential to identify a viable avenue for treatment of disease pathology, potentially toward practical near-term therapies for RTT.

### CNS-specific mis-regulation of Irak1 in Mecp2-null mice

**Figure 1:**
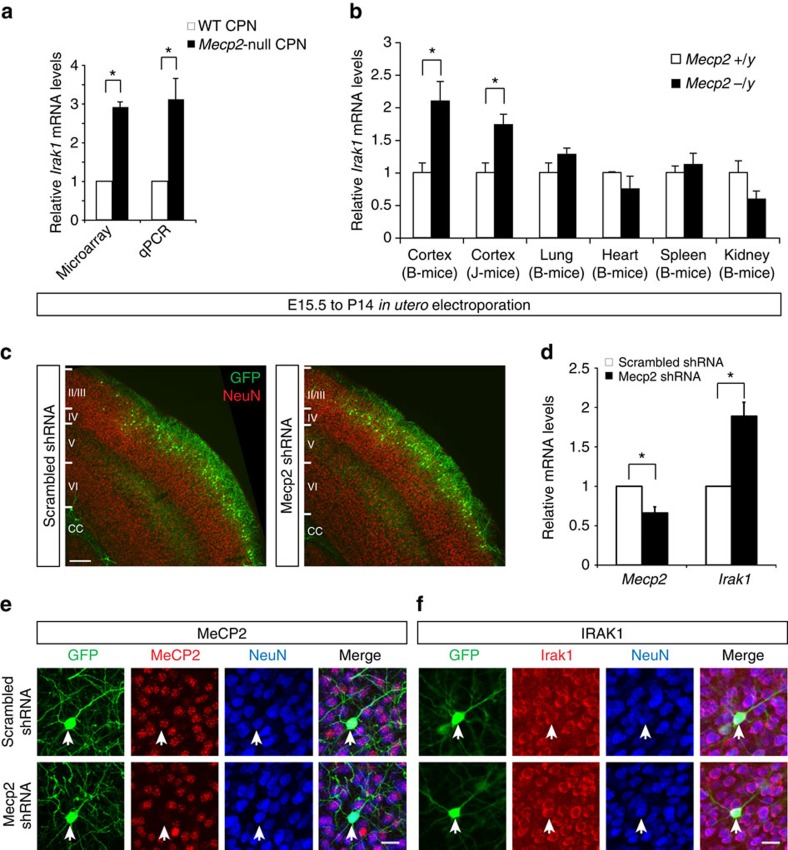
MeCP2 represses *Irak1* expression in a brain-specific manner. (**a**) In our microarray analyses, three independent probe sets reveal that *Irak1* is overexpressed an average of 2.9-fold in P14 *Mecp2*-null CPN, which was confirmed by qPCR on three independent biological replicates. *P<0.05, **P<0.01, paired two-sided *t*-test. (**b**) qPCR at 6 weeks of age reveals that *Irak1* is overexpressed in cortices of two independent *Mecp2*-null mouse lines (B-mice and J-mice), when compared with wild-type cortex, but not in multiple non-CNS tissues. *P<0.05, unpaired two-sided *t*-test, mean±s.e.m. (**c**–**f**) To exclude the possibility that overexpression of *Irak1* is an artefact of gene targeting of *Mecp2*, we knocked down *Mecp2* in wild-type layer 2/3 neurons by *in utero* electroporation of an *Mecp2* shRNA, in comparison with a control shRNA, at E15.5. (**c**) Cells electroporated with *Mecp2* shRNA do not exhibit any disruptions in migration, laminar location or survival in comparison to control, scrambled shRNA, as indicated by GFP reporter expression. Scale bar, 200 μm. (**d**) Electroporated cells were purified by FACS at P14, and qPCR analysis reveals that *Irak1* is overexpressed an average of 1.9-fold following an approximate 40% reduction in *Mecp2* mRNA. *P<0.05 from three independent experiments, paired two-sided *t*-test. (**e**) MeCP2 protein (red) is detected in P14 layer 2/3 neurons (NeuN, blue) electroporated with a control, scrambled shRNA, but it is not detected in layer 2/3 neurons electroporated with an *Mecp2* shRNA construct. (**f**) IRAK1 protein (red) is detected in layer 2/3 NeuN positive (blue) neurons electroporated with either scrambled or *Mecp2* shRNA. Scale bar, 25 μm (**e**,**f**).

Three independent probe sets identified approximately 3-fold overexpression of *Irak1* in the initial microarray analysis, and we validated these findings using quantitative real-time PCR (qPCR; Fig. 1a). Interestingly, a number of other studies have also found overexpression of *Irak1* in *Mecp2*-null brain regions^28^, but *Irak1* has not previously been biologically investigated as an MeCP2 target gene. We confirmed that *Irak1* expression is aberrantly increased in the cortex of two independent lines of *Mecp2*-null mice, in which the *Mecp2* locus was differently targeted^4^,^5^ (Fig. 1b). Next, to investigate whether *Irak1* overexpression is specific to the *Mecp2*-null CNS, or whether it is present in all organ systems, we analysed *Irak1* expression levels in multiple distinct organs of *Mecp2*-null mice, including lung, heart, spleen and kidney, in addition to the cerebral cortex. This analysis reveals that the increase in expression of *Irak1* is, indeed, specific to *Mecp2*-null cortex, with no significant change in non-neural tissues examined (Fig. 1b).

Further, we assessed whether shRNA-mediated knockdown of *Mecp2* similarly upregulates *Irak1* expression in otherwise wild-type CPN. We electroporated either a scrambled control or *Mecp2* shRNA construct into cortical progenitors at E15.5, the peak of layer 2/3 CPN generation, and purified electroporated cells at P14 via FACS. For these studies, we employed an shRNA targeting *Mecp2* that has been previously thoroughly validated to be effective and specific^23^,^28^,^29^. In agreement with previous studies employing these shRNA constructs, shRNA-mediated knockdown of *Mecp2* does not appear to disrupt migration or survival of layer 2/3 CPN^23^ (Fig. 1c). Analysis by qPCR reveals a modest ∼35% knockdown of *Mecp2* at the mRNA level (Fig. 1d), while immunocytochemistry demonstrates highly reduced MeCP2 protein detection following knockdown (Fig. 1e), in keeping with previous studies^23^,^29^. This reduction of MeCP2 expression by wild-type CPN is sufficient to increase *Irak1* expression approximately 2-fold (Fig. 1d). We confirmed by immunocytochemistry that IRAK1 protein is, indeed, expressed by layer 2/3 CPN (Fig. 1f); however, the inherent limitations and variability of immunocytochemistry, combined with the decreased soma size and increased packing density of *Mecp2*-null CPN^19^, prevent reliable quantitative assessment of IRAK1 protein following only a 2–3-fold upregulation of *Irak1* mRNA with *Mecp2* loss-of-function.

The *Irak1/IRAK1* locus is located ∼10 kb downstream of the *Mecp2/MECP2* locus in both mouse and human genomes (Supplementary Fig. 2), raising the theoretical possibility that genome modification of the *Mecp2* locus artifactually upregulates *Irak1* expression because of this proximity of the loci. However, taken together, our experiments confirm that overexpression of *Irak1* in *Mecp2*-null cortex results from loss of *Mecp2* function specifically in the CNS; it is not an artefact of the gene targeting of the *Mecp2* locus.

A recent study reported that MeCP2 can repress *Irak1* expression via mirR-146a (ref. 30). To determine whether MeCP2 might also directly regulate *Irak1* expression in the cortex, we investigated whether MeCP2 binding sites exist on the *Irak1* promoter. We first analysed the methylation status of CpGs around the transcription initiation site of *Irak1* via bisulfite genomic sequencing (Supplementary Fig. 3). While the majority of CpGs on or near exons 1 and 2 are methylated at very low levels, consistent with a previous study^31^, five CpGs in a region upstream of *Irak1* (−2,107; −1,492; −779; −547; and −426 bp) are highly methylated, and chromatin immunoprecipitation (ChIP) analysis identifies that MeCP2 binds specifically to the CpG at −1,492 bp upstream of the *Irak1* transcription initiation site (Supplementary Fig. 3). These results indicate that *Irak1* expression in the cortex might be regulated both directly and indirectly by MeCP2. This potential dual mode of regulation of *Irak1* by MeCP2, and the identification of *Irak1* upregulation in multiple *Mecp2*-null brain regions (including cortex, hippocampus, and cerebellum) by multiple laboratories, indicates that *Irak1* is an important MeCP2 target gene, requiring tight regulation in neurons. We, therefore, investigated whether aberrant overexpression of *Irak1* might underlie, at least in part, *Mecp2*-null neuronal phenotypes.

### Overexpression of Irak1 disrupts CPN dendritic complexity

**Figure 2:**
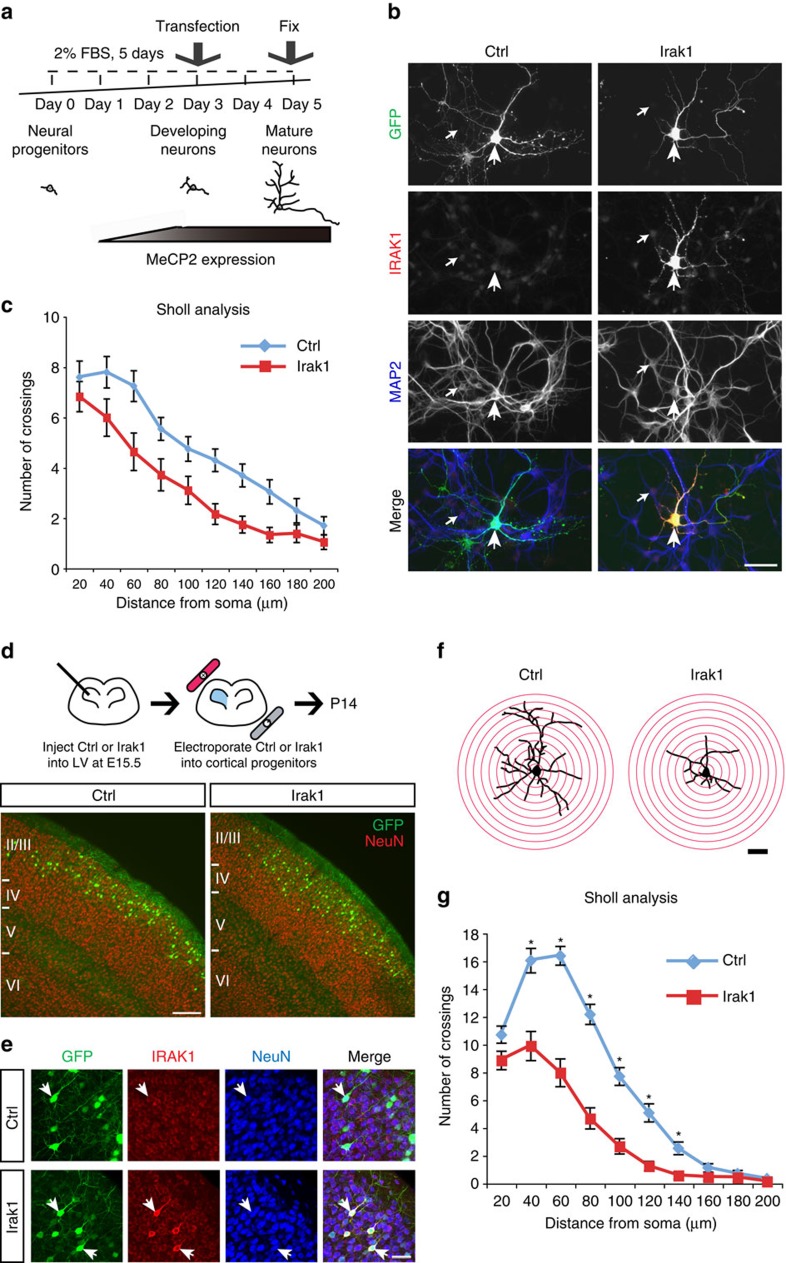
Overexpression of *Irak1* in cortical neurons reduces dendritic arborization. (**a**) A plasmid overexpressing either a reporter GFP (Ctrl) or both *Irak1* and GFP (Irak1) was transfected into developing immature neurons in culture. (**b**) IRAK1 protein (red) is highly expressed by neurons (MAP2 positive; blue) transfected with *Irak1* and GFP (green), while neurons transfected with GFP alone express only endogenous, low levels of IRAK1. Large arrowheads indicate transfected neurons; small arrows indicate neighbouring, untransfected neurons. Scale bar, 50 μm. (**c**) Cells were immuno-labelled against both MAP2 and GFP, and the dendritic morphology of MAP2+/GFP+ cells was analysed by Sholl analysis. Overexpression of *Irak1* significantly decreases neuronal dendritic arborization, compared to that of control neurons (control *n*=20, *Irak1*-overexpressing neurons *n*=36); two-way ANOVA, F(1,540)=39.9, *P*<0.001). Error bars=mean±s.e.m. (**d**) To investigate whether overexpression of *Irak1* also modifies dendritic complexity of layer 2/3 neurons *in vivo*, Ctrl and Irak1 plasmids were injected *in utero* into the lateral ventricle (LV), and electroporated into neural precursors in the ventricular zone (VZ) at E15.5. Electroporated neural precursors subsequently give rise to differentiated cortical layer 2/3 projection neurons (NeuN+, red), with no obvious disruption in laminar location or survival at P14 with *Irak1* overexpression. Scale bar, 200 μm. (**e**) IRAK1 protein (red) is highly overexpressed by P14 layer 2/3 neurons electroporated with Irak1, but not Ctrl, compared to endogenous IRAK1 expression within cortical layer 2/3 at P14. Scale bar, 50 μm. (**f**,**g**) We analysed the dendritic morphology of electroporated layer 2/3 projection neurons at P14 by Sholl analysis (ctrl n=24, Irak1 *n*=28). *In vivo* overexpression of *Irak1* in layer 2/3 projection neurons results in reduced dendritic arborization, recapitulating the dendritic phenotype in layer 2/3 projection neurons in *Mecp2*-null mice. *P<0.01, a two-way ANOVA and the Bonferroni test, mean±s.e.m., Scale bars, 50 μm.

To determine whether overexpression of *Irak1* induces the reduced dendritic complexity phenotype of *Mecp2*-null CPN, we first overexpressed *Irak1* in developing immature cortical neurons in culture; these neural progenitor cultures largely recapitulate *in vivo* neuronal differentiation and maturation (Fig. 2a). In these experiments, IRAK1 protein is very highly overexpressed by transfected neurons, compared with endogenous expression by neighbouring untransfected neurons, or those transfected with a green fluorescent protein (GFP) only control (Fig. 2b). We visualized the morphology of individual transfected neurons using GFP reporter fluorescence, and analysed the effects of overexpression of *Irak1* on dendritic arborization by Sholl analysis (Fig. 2c). Our results indicate that overexpression of *Irak1* in developing immature cortical neurons decreases dendritic complexity, strikingly mimicking the phenotype we previously reported in *Mecp2*-null CPN^19^,^20^.

To determine whether *Irak1* has a similar function *in vivo*, we introduced either a control GFP-construct or an *Irak1*-GFP construct into cortical neural progenitors at E15.5 (Fig. 2d,e), the time of peak generation of neocortical layer 2/3 neurons, then visualized and analysed the dendritic morphology of GFP-labelled electroporated layer 2/3 projection neurons by Sholl analysis at P14 (Fig. 2f,g). For these experiments, we employed a construct with *Irak1* driven by a *tau* promoter, to overexpress *Irak1* post-mitotically and avoid potential disruption at the progenitor level. We confirmed that there is no overt disruption of CPN differentiation, laminar position or survival with *Irak1* overexpression (Fig. 2d), and that IRAK1 protein is highly overexpressed by electroporated layer 2/3 pyramidal neurons, compared with endogenous expression by either neighbouring non-electroporated neurons, or those electroporated with the GFP-only control (Fig. 2e). Consistent with the *in vitro* experiments, overexpression of *Irak1* by layer 2/3 pyramidal neurons *in vivo* leads to reduced dendritic arborization, confirming that overexpression of *Irak1* closely recapitulates the dendritic phenotype of *Mecp2*-null mice. Exogenous overexpression of *Irak1* drives expression at a much higher level than the 3-fold overexpression of *Irak1* by *Mecp2*-null CPN. Interestingly, this enhanced IRAK1 upregulation leads to a dendritic phenotype by P14 that is strikingly equivalent to that we previously reported only in adult *Mecp2*-null mice^19^,^20^, suggesting that the level of IRAK1 overexpression could alter phenotypic progression.

### NF-κB signalling is upregulated with Irak1 overexpression

**Figure 3:**
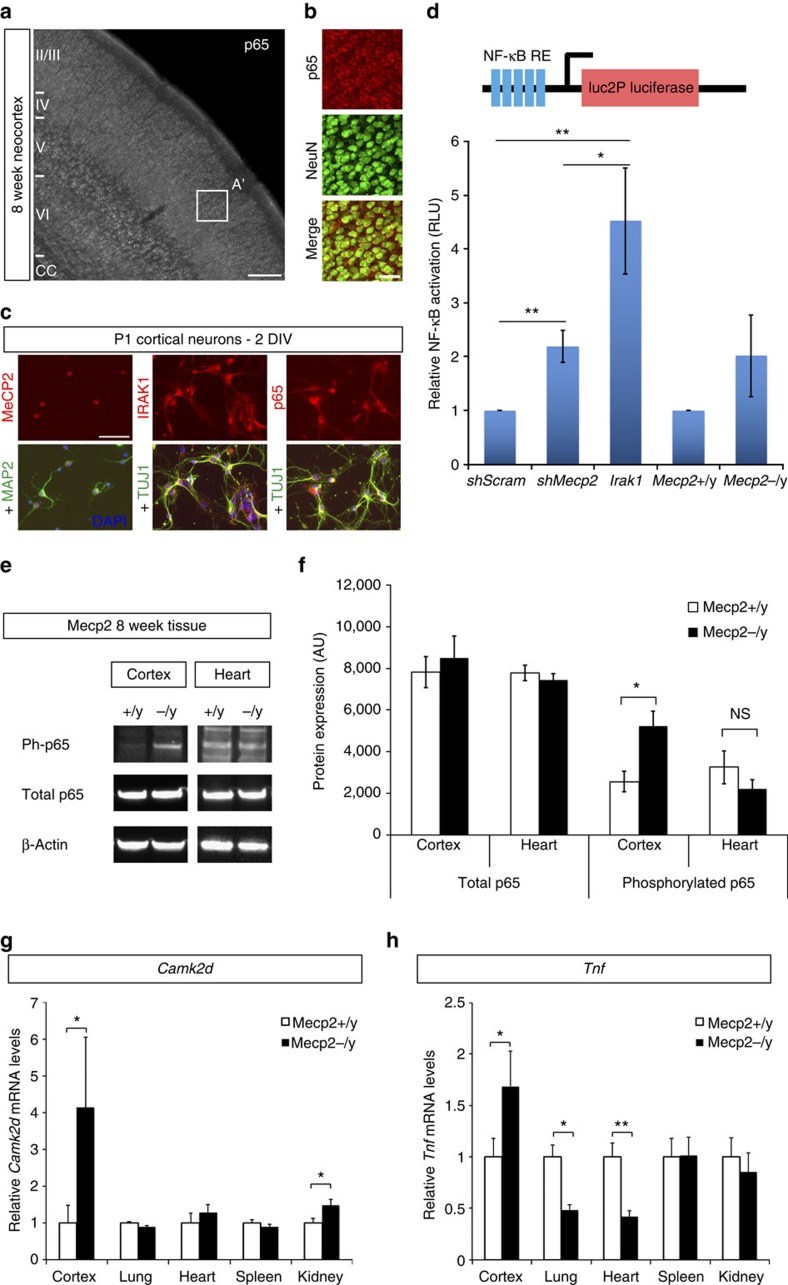
NF-κB signalling is upregulated by cortical neurons with *Mecp2* loss-of-function. (**a**,**b**) The NF-κB subunit p65 (also known as RelA; grey in **a**, red in **b**) is widely expressed by neurons throughout the neocortex (NeuN positive, green), including CPN in layer 2/3. Scale bar, 200 μm. (**c**,**d**) We directly investigated whether MeCP2 and IRAK1 regulate NF-κB signalling in cortical neurons. (**c**) We confirmed that wild-type P1 neurons express MeCP2, IRAK1 and p65/RelA (all red), with a primarily cytoplasmic (inactive) localization at 2 days *in vitro* (DIV), as well as the neuronal markers MAP2 and TUJ1 (green). Scale bar, 50 μm. (**d**) We nucleofected P1 cortical neurons with an NF-κB luciferase reporter construct, and either a control shRNA construct, an *Mecp2* shRNA construct, or an *Irak1* expression construct, and measured NF-κB activity by luciferase assay at 2 DIV (*n*=5 independent experiments). Knockdown of *Mecp2* results in an approximate 2.2-fold increase in activation of NF-κB over control, while overexpression of *Irak1* increases NF-κB activation 4.5-fold on average. We additionally compared NF-κB luciferase reporter activity in P1 *Mecp2* wild-type and *Mecp2*-null cortical neurons, and identified a trend towards increased NF-κB activation in *Mecp2*-nulls (*n*=3 independent experiments). RLU, relative luminescence units, normalized to a control luciferase construct. *P<0.05, **P<0.01, unpaired two-sided *t*-test, mean±s.e.m. (**e**,**f**) We investigated NF-κB activation *in vivo* in 8 week neocortex and heart by western blot for phosphorylated p65/RelA, relative to total p65/RelA, and (**e**) identified an approximately 2-fold increase in phosphorylated p65/RelA specifically in *Mecp2*^−/y^ cortex compared with littermate *Mecp2*+/y (*n*=3 littermate pairs). AU, arbitrary units. *P<0.05, unpaired two-sided *t*-test, mean±s.e.m. (**g**,**h**) Further, qPCR experiments using mRNA from wild-type and *Mecp2*-null mice (four littermate pairs) at 8 weeks of age reveal that two downstream genes involved in NF-κB signalling, *Camk2d* (**g**) and *Tnf* (**h**), are upregulated in cortex, but are not widely upregulated in non-CNS tissues. *Gapdh* expression was used as an internal control. *P<0.05, **P<0.001, unpaired two-sided *t*-test, mean±s.e.m.

Because IRAK1 is known to function as a signalling kinase and scaffolding protein within the NF-κB signalling pathway, we hypothesized that the dysregulated overexpression of *Irak1* by *Mecp2*-null CPN modifies dendritic complexity, at least in part, through aberrant NF-κB activation. NF-κB signalling is known to regulate neural process development and structural plasticity, including dendritic complexity (reviewed in Gutierrez and Davies^27^). NF-κB is a ubiquitously expressed transcription factor, composed of homodimers or heterodimers of a family of five proteins; it is held in an inactive form in the cytoplasm, but is released by a variety of extracellular signals, allowing it to translocate to the nucleus, and bind to consensus NF-κB response elements to activate transcription of target genes. The predominant form of NF-κB in the nervous system is a p65/p50 heterodimer^27^; we confirmed that p65 (also known as RelA) is expressed in the cytoplasm of neurons throughout the neocortex, including layer 2/3 CPN (Fig. 3a,b).

To directly investigate whether MeCP2 and IRAK1 regulate NF-κB signalling in cortical neurons, we employed a reporter construct with tandem NF-κB-response elements and a minimal reporter driving luciferase to assay NF-κB transcriptional activity, after confirming that MeCP2, IRAK1 and p65/RelA are expressed by the dissociated postnatal neocortical neurons (Fig. 3c). Knocking down *Mecp2* via shRNA results in an approximately 2-fold increase in NF-κB activity over a control shRNA, while overexpressing *Irak1* (resulting in IRAK1 expression that is much higher than the physiological 2–3-fold increase observed following *Mecp2* loss-of-function) increases NF-κB activity almost 5-fold (Fig. 3d). We further analysed NF-κB transcriptional activity in cultured *Mecp2*-null and wild-type littermate cortical neurons, and, consistent with all the results above, identified a trend toward 2-fold increase in NF-κB activity with *Mecp2* loss-of-function (Fig. 3d).

**Figure 4:**
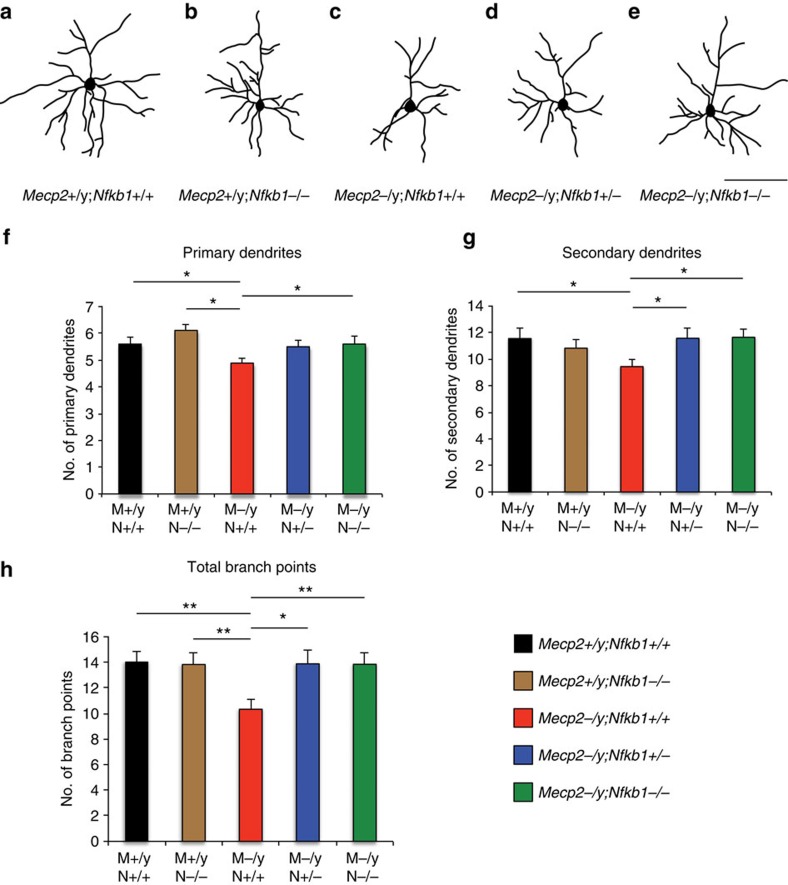
Reducing NF-κB signalling improves reduced dendritic complexity of *Mecp2*-null CPN. To determine whether rough correction of abnormally activated NF-κB signalling due to overexpression of *Irak1* can partially rescue the reduced dendritic arborization in *Mecp2*-null cortex, we generated *Mecp2* and *Nfkb1* double mutant mice, and visualized neuronal morphology by Golgi staining. Representative examples of camera lucida drawings of layer 2/3 pyramidal neurons in *Mecp2*^+/y^;*Nfkb1*^+/+^ (**a**, *n*=30); *Mecp2*^+/y^; *Nfkb1*^−/−^ (**b**, *n*=18); *Mecp2*^−/y^;*Nfkb1*^+/+^ (**c**, *n*=18), *Mecp2*^−/y^;*Nfkb1*^+/−^ (**d**, *n*=26); and *Mecp2*^−/y^; and *Nfkb1*^−/−^ (**e**, *n*=20) mice at 8 weeks of age. The numbers of primary (**f**) and secondary dendrites (**g**), and total branch points (**h**) in *Mecp2*^−/y^;*Nfkb1*^+/−^ and *Mecp2*^−/y^;*Nfkb1*^−/−^ neurons are rescued (increased) compared with those of *Mecp2*^−/y^;*Nfkb1*^+/+^ neurons. *P<0.05, **P<0.01 unpaired two-sided *t*-test, mean±s.e.m. Scale bar, 100 μm (**a**–**d**).

To confirm that NF-κB activity is aberrantly increased in the *Mecp2*-null cortex *in vivo*, we employed phosphorylation of p65/RelA as an indication of NF-κB activation (Fig. 4e)^32^. There is an approximately 2-fold increase in the relative quantity of phosphorylated p65/RelA in 8-week-old *Mecp2*-null cortex compared with littermate controls, while phosphorylated p65/RelA is not significantly different in *Mecp2*-null heart (Fig. 3e,f) where *Irak1* is not upregulated (Fig. 1b). We further analysed the expression of two genes regulated by NF-κB signalling, *Camk2d* and *Tnf*^26^,^33^. These genes are overexpressed in *Mecp2*-null 8-week-old cortex, but are not widely upregulated in multiple *Mecp2*-null non-CNS tissues (Fig. 3g,h), where *Irak1* is also not upregulated (Fig. 1b). Together, these results further support the conclusion that abnormal activation of NF-κB signalling in *Mecp2*-null cortex is correlated with perturbed regulation of *Irak1* expression, and is specific to the CNS.

### Attenuation of NF-κB signalling rescues dendritic complexity

To investigate the hypothesis that modulation of NF-κB signalling might partially rescue dendritic complexity of *Mecp2*-null layer 2/3 pyramidal neurons, we crossed *Mecp2* mutant mice with *Nfkb1* mutant mice. *Nfkb1*, encoding the p50 subunit of the DNA-binding NF-κB complex, is located on mouse chromosome 3, and is genetically downstream of *Irak1* in NF-κB signalling. We visualized the dendritic morphology of *Mecp2*^+/y^;*Nfkb1*^+/+^ and *Mecp2*^+/y^;*Nfkb1*^−/−^ and *Mecp2*^−/y^;*Nfkb1*^+/+^ and *Mecp2*^−/y^;*Nfkb1*^+/−^ and *Mecp2*^−/y^; *Nfkb1*^−/−^ layer 2/3 pyramidal neurons by Golgi staining (Fig. 4a–e). While an initial (relatively low sensitivity) screen of dendritic complexity by Sholl analysis does not detect significant differences in *Mecp2*-null dendritic complexity with additional disruption of *Nfkb1* (Supplementary Fig. 4), a more sensitive analysis of dendritic complexity reveals that the numbers of primary (Fig. 4f) and secondary dendrites (Fig. 4g), and total branch points (Fig. 4h), in *Mecp2*^−/y^;*Nfkb1*^+/−^ and *Mecp2*^−/y^;*Nfkb1*^−/−^ neurons are substantially rescued (increased) compared with those of *Mecp2*^−/y^;*Nfkb1*^+/+^ neurons. While Sholl analysis detects two-dimensional extension of neuronal dendrites comprehensively, it often overlooks more subtle, but functionally important, differences in fundamental components, such as the number of primary and secondary dendrites. Importantly, there is no significant difference in complexity with loss of *Nfkb1* in *Mecp2*^+/y^ mice; this indicates that loss of *Nfkb1* function alone does not enhance dendritic arborization of adult layer 2/3 pyramidal neurons *in vivo*, independent of *Mecp2* loss-of-function. Taken together, these results indicate that abnormal activation of NF-κB signalling critically contributes to the reduced dendritic complexity in *Mecp2*-null CPN.

### Disrupted NF-κB contributes to pathogenesis of Mecp2-nulls

**Figure 5:**
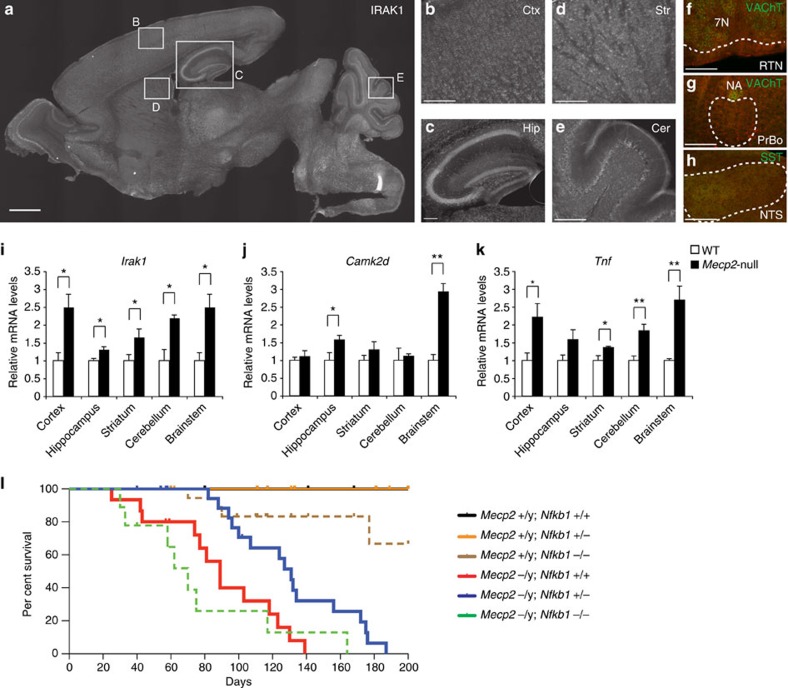
Reducing aberrant NF-κB signalling improves lifespan of *Mecp2*-null mice. (**a**) IRAK protein is expressed by neurons widely throughout the P14 brain, including in the (**b**) neocortex, (**c**) hippocampus, (**d**) striatum and (**e**) cerebellum. (**f**,**h**) IRAK1 (red) is also expressed in many brainstem regions that are critical for respiratory function, including (F) the retrotrapezoidal nucleus (RTN), located ventral to the VAChT-positive (green) facial nucleus (7N); (**g**) the pre-Botzinger complex (PrBo), located ventral to the VAChT-positive (green) nucleus ambiguus; and (**h**) the somatostatin (SST; green) positive nucleus of the solitary tract (NTS). Scale bar, 1 mm (**a**); scale bar, 200 μm (**b**–**h**). (**i**–**k**) RNA was isolated from cortex, hippocampus, striatum, cerebellum and brainstem of P14 wild-type (*n*=3) and *Mecp2*-null mice (*n*=3). Expression levels of *Irak1* (**i**) and downstream genes involved in NF-κB signalling, *Camk2d* (**j**) and *Tnf* (**k**), were analysed by qPCR, and normalized to the expression level of *Gapdh*. These genes are widely dysregulated in multiple brain regions, including the cortex and the brainstem (implicated in respiratory dysfunction in RTT). *P<0.05, **P<0.01, unpaired two-sided *t*-test, mean±s.e.m. (**l**) Kaplan–Meier survival curves for *Mecp2*^+/y^; *Nfkb1*^+/+^ (black line, *n*=21), *Mecp2*^+/y^; *Nfkb1*^+/−^ (orange line, *n*=28), *Mecp2*^+/y^; *Nfkb1*^−/−^ (brown line, *n*=19), *Mecp2*^−/y^; *Nfkb1*^+/+^ (red line, *n*=15), *Mecp2*^−/y^; *Nfkb1*^+/−^ (blue line, *n*=21); and *Mecp2*^−/y^; *Nfkb1*^−/−^ (green line, *n*=9). Log-rank survival analysis reveals that *Mecp2*^−/y^; *Nfkb1*^+/−^ mice survive significantly and substantially longer to blinded standardized humane morbidity criteria (∼50% increase) than *Mecp2*^−/y^; *Nfkb1*^+/+^ mice (*P*=0.002; median survival 131 versus 89 days, respectively).

Because RTT pathology is far from limited to reduced CPN dendritic complexity, we investigated whether dysregulation of *Irak1* and NF-κB signalling occurs more broadly in the brain, and might function in other pathological phenotypes of *Mecp2*-null mice. Similar to the widespread expression of MeCP2 across the brain, IRAK1 is present in neuronal populations throughout the brain (Fig. 5a), including the neocortex, hippocampus, striatum and cerebellum (Fig. 5b–e). IRAK1 is also present throughout the pons and medulla, including (but not limited to) brainstem nuclei that regulate respiratory function and play a central role in autonomic dysfunction and lifespan in *Mecp2*-null mice^16^. These include the retrotrapezoidal nucleus, pre-Botzinger complex and nucleus of the solitary tract (Fig. 5f–h). The same widespread expression of IRAK1 is observed in *Mecp2*-null brains (data not shown), and qPCR analysis using RNA from P14 wild-type and *Mecp2*-null tissue confirms that *Irak1* is overexpressed broadly in *Mecp2*-null brain: in hippocampus, cerebellum and brainstem, as well as in neocortex (Fig. 5i). We further analysed two genes regulated by NF-κB signalling, *Camk2d* and *Tnf*^26^,^33^, in these brain regions. Already at this early symptomatic stage (P14), *Tnf* is overexpressed broadly throughout the *Mecp2*-null brain, with upregulation of *Camk2d* in several regions (Fig. 5j,k), confirming that NF-κB signalling is abnormally activated across multiple areas of the *Mecp2*-null CNS.

Finally, to examine whether this brain-wide abnormal activation of NF-κB signalling contributes to broader aspects of the pathogenesis of *Mecp2*-null mice, in addition to reduction in CPN dendritic complexity, we analysed the lifespan of double *Mecp2* and *Nfkb1* mutant mice. Early adult lethality is a common phenotype in multiple lines of *Mecp2*-null mice^4^,^5^; lifespan is dependent on MeCP2 function within HoxB1-derived tissues of the brainstem and spinal cord^16^, and cannot be rescued by restoring MeCP2 function in the forebrain alone^14^. Further, increased NF-κB signalling underlies the early lethality of *Sirt6* mutant mice, and that phenotype can be rescued by correction of aberrantly increased NF-κB signalling^34^, suggesting that abnormally increased NF-κB signalling in the brainstem of *Mecp2*-null mice might contribute to their shortened lifespan.

We found that, while *Mecp2*^−/y^;*Nfkb1*^+/+^ mice reach reproducible, institutionally mandated (and fully blinded) criteria for euthanasia at an average of 89 postnatal days in our colony (red line in Fig. 5l), the average lifespan for *Mecp2*^−/y^;*Nfkb1*^+/−^ mice extends to 131 days (blue line), demonstrating that a reduced, 50% expression level of *Nfkb1* in *Mecp2*-null mice substantially improves health and thus extends the lifespan of *Mecp2*-null mice by ∼50% (log-rank test, *P*=0.0021). The lifespan of *Mecp2*^−/y^;*Nfkb1*^−/−^ mice (green line) is substantially shortened (median survival 70 days; log-rank test, *P*=0.0035) compared with that of *Mecp2*^−/y^;*Nfkb1*^+/−^ mice. Although *Nfkb1*^−/−^ mice are generally recognized to be viable, they are more prone to infection, and die more frequently at an earlier age^35^ (regardless of *Mecp2* status). Consistent with this, we also observed premature death of *Mecp2*^+/y^;*Nfkb1*^−/−^ mice (brown line), while no *Mecp2*^+/y^;*Nfkb1*^+/+^ or *Mecp2*^+/y^;*Nfkb1*^+/−^ mice (black and orange lines, respectively) died before 200 days. Therefore, increased susceptibility to infection due to complete loss of *Nfkb1* function might likely reduce the lifespan of *Mecp2*^−/y^;*Nfkb1*^−/−^ mice. Taken together, these data indicate that correction of NF-κB signalling to roughly appropriate levels, without complete loss of NF-κB signalling, strikingly ameliorates both aberrant dendritic complexity in the neocortex, and *Mecp2*-null postnatal mortality.

## Discussion

In these experiments, we identify increased, aberrant expression of *Irak1* caused by *Mecp2* loss-of-function, and reveal that the resulting abnormal activation of NF-κB signalling is critically involved in the pathogenesis of *Mecp2*-null mice. While NF-κB signalling has been most highly studied, and is best understood, in the immune system, there is an extensive literature investigating the regulation of neural process development and structural plasticity by NF-κB, including dendritic complexity, in addition to implicating NF-κB regulation in learning and memory (reviewed in Gutierrez and Davies^27^). NF-κB subunits are expressed throughout the CNS, by neurons as well as by glia. Within neurons, NF-κB subunits are detected in neuronal processes and synapses, signalling back to the nucleus in response to a variety of extracellular stimuli, including Ca^2+^, neurotransmitters, neuropeptides, neurotrophins, cytokines and neural cell adhesion molecules. NF-κB signalling either promotes or inhibits neurite growth in a context-dependent manner, with neuronal subtype and developmental stage being two key variables^26^,^27^,^36^,^37^. In fact, a developmental switch in NF-κB signalling is necessary for neurite outgrowth^36^, and NF-κB activity increases during neuronal differentiation^38^. Further, NF-κB signalling can either promote or inhibit neurite growth in the same neurons, depending on the mechanisms of NF-κB activation^37^. This highly dynamic developmental regulation of NF-κB likely results in the reduced dendritic complexity observed in embryonic cortical neurons cultured from *Nfkb1*-null mice^39^, while there is no disruption in dendritic complexity in adult *Nfkb1*-null CPN on an *Mecp2* wild-type background (Fig. 4). Thus, even modest dysregulation of the activity of this key signalling pathway might lead to widespread perturbations of neural development and function.

Recently, null mutations in CC2D1A, a scaffolding protein that regulates multiple effectors upstream of NF-κB, were found to cause a spectrum of cognitive phenotypes, including intellectual disability and autism spectrum disorders^40^. Knockdown of *Cc2d1a* in mouse hippocampal neurons *in vitro* results in reduced dendritic complexity, which can be rescued by NF-κB inhibitors. Further, mutations in TRAPPC9, which encodes a binding protein for NF-κB-inducing kinase and IκB kinase complex beta, have been identified in multiple families with intellectual disability^41^,^42^,^43^. These findings, taken together with our results, strongly suggest that excessive NF-κB signalling disturbs normal brain function via aberrant circuit assembly, and that the neurodevelopmental disorder RTT (and potentially some autism spectrum disorders and other intellectual disabilities) is in consequential part attributable to abnormal activation of NF-κB signalling.

Our results also offer new insight into a neurodevelopmental disorder that partially displays RTT-like symptoms, and is caused by duplication of a genomic region that includes the *MECP2* locus. This duplication is thought to cause ∼1% of unexplained X-linked cognitive disability^1^,^44^. While the neurodevelopmental abnormalities have been attributed to gene dysregulation caused by increased *MECP2* expression^45^,^46^, *MECP2* and *IRAK1* are both included in the duplicated regions of all patients with this disorder examined to date^44^. Given our findings that overexpression of *Irak1* significantly disrupts cortical neuronal dendritic morphology (Fig. 2), it seems likely that duplication of *IRAK1* itself, with associated increase in its expression, might partially and directly contribute to the pathogenesis of this category of cognitive disability, potentially explaining why RTT and X-linked cognitive disability due to duplication of *MECP2* share similar symptoms.

Common regulation pathways shared by the immune system and nervous system have increasingly been identified in the past decade. In particular, microarray analyses of human autistic brain tissue have detected increased transcript levels of many genes standardly viewed as immune system-related, including components of the NF-κB signalling pathway^47^, and genome-wide association studies of multiple psychiatric disorders implicate both (what are standardly considered) neuronal and immune pathways^48^. Further, maternal immune activation through interleukin 6 (IL-6), a downstream target of NF-κB signalling, results in offspring that display behaviours linked to schizophrenic and autistic behaviour in rodents^49^, and *Mecp2* knockdown *in vitro* in myeloid lineage cells can enhance NF-κB signalling and increase expression of inflammatory cytokines^50^,^51^. A recent report indicates that microglia, the resident immune cells in the CNS, play a critical role in RTT^52^, and microglia-neuron immune crosstalk in the hypothalamus can regulate longevity via NF-κB signalling^32^. It is unlikely that this microglial mechanism underlies the increased longevity of *Mecp2*^−/y^;*Nfkb1*^+/−^ mice, however. Reducing NF-κB signalling (*Nfkb1*^+/−^) increases lifespan from ∼90 to ∼130 days in *Mecp2*-null mice, but has no effect on *Mecp2* wild-type mice, within the timeframe of our analyses (Fig. 5). Genetically attenuating NF-κB signalling in microglia of the hypothalamus, on the other hand, only alters aging phenotypes in old mice (greater than 18 months), and a CNS-specific knockdown of NF-κB signalling (*Ikbkb*^Fl/Fl^) extends average lifespan from ∼800 to ∼1,000 days^32^.

Based on findings that reactivation of MeCP2 can reverse neurological phenotypes and lethality of *Mecp2*-null mice^53^,^54^, even at advanced symptomatic stages, attempts have been made to improve the developmental deficits of *Mecp2*-null mice, towards future therapeutic approaches for RTT. While some recent studies have focused on MeCP2 functions as a global inhibitor of transcriptional noise^8^,^55^,^56^, other recent studies indicate that modification of transcription of a single MeCP2 target gene or pathway can improve phenotypes of *Mecp2*-null mice. For example, overexpression of *Bdnf* in *Mecp2*-null mice^57^; treatment with statin drugs to correct aberrant cholesterol homeostasis in *Mecp2*-null mice^58^; and systemic treatment of *Mecp2*-null mice with IGF1 (refs. 59,6059,60), can all partially ameliorate the neurological abnormalities and extend lifespan. In addition, treatment of female heterozygous mice with a TrkB agonist can restore wild-type breathing frequency^61^.

Because NF-κB signalling has been extensively investigated, and because many inhibitors of this pathway have been identified and are already in use clinically—including vitamin D, disulfram and sodium salicylate, our results strongly suggest that modulation of NF-κB signalling via relatively modest pathway inhibition might provide a viable avenue for treatment of disease pathology, potentially towards practical near-term therapies for RTT. The known ability of vitamin D and its analogues to inhibit NF-κB signalling^62^,^63^ is particularly compelling given the high prevalence of vitamin D deficiency in RTT patients^64^. Developmental vitamin D deficiency leads to severe neurodevelopmental disruptions and behavioural abnormalities in rodents (reviewed in Eyles *et al*.^65^), and there is growing evidence of a correlation between vitamin D deficiency and autism spectrum disorders^66^,^67^,^68^. The precise mechanisms by which vitamin D regulates neurodevelopment are not known, but these findings raise interesting questions regarding converging underlying mechanisms and possible involvement of NF-κB signalling.

Taken together, our results indicate that abnormal activation of NF-κB signalling, at least in part via loss of *Mecp2* regulation of *Irak1* expression, is critically involved in RTT-like cerebral cortex dysgenesis, reduced dendritic-circuit complexity, and reduced health, function and lifespan of *Mecp2*-null mice. These results provide new insight into both the fundamental neurobiology of RTT, and potential therapeutic strategies via NF-κB pathway modulation.

## Methods

### Animals

All animal experimental protocols were approved by the Massachusetts General Hospital and / or Harvard University Institutional Animal Care and Use Committee, and adhere to NIH guidelines. Mice were group housed at the maximum of five mice per cage on a 12:12 h light/dark cycle, and were given food and water *ad libitum*. Wild-type C57BL/6 and CD1 mice were purchased from Charles River Laboratories (Wilmington, MA). Female *Mecp2* heterozygous mice were generously provided by Dr Adrian Bird (B-mice)^5^, and were maintained on a C57BL/6 background. A second line of female *Mecp2* heterozygous mice (mixed background; 129, C57BL/6, and BALB/c) were generously provided by Dr Rudolf Jaenisch (J-mice)^4^, and backcrossed into a C57BL/6 background for a minimum of 10 generations. Male *Nfkb1* homozygous mice (B6.Cg-*Nfkb1*^*tm1Bal*^/J) were purchased from the Jackson laboratory, and were backcrossed to C57BL/6 for 12 generations (Bar Harbor, ME). The age and sex of mice used are described in the methods for each experiment. The genotypes of the three lines of mice were determined by PCR on tail genomic DNA as follows:

*Mecp2* mutant mice (B-mice)—forward primer oIMR1436 5′- GGTAAA GAC CCA TGT GAC CC -3′; reverse primer oIMR1437 5′- TCC ACC TAG CCT GCC TGT AC -3′; reverse primer oIMR1438 5′- GGC TTG CCA CAT GAC AA -3′.

*Mecp2* mutant mice (J-mice)—forward primer Nsi-5 5′- CAC CAC AGA AGT ACT ATG ATC -3′; 2lox-3 5′- CTA GGT AAG AGC TCT TGT TGA -3′; Nsi-3 5′- ATG CTG ACA AGC TTT CTT CTA -3′.

*Nfkb1* mutant mice—forward primer oIMR0476 5′- GCA AAC CTG GGA ATA CTT CAT GTG ACT AAG -3′; reverse primer oIMR0477 5′- ATA GGC AAG GTC AGA ATG CAC CAG AAG TCC -3′; reverse primer oIMR0478 5′- AAA TGT GTC AGT TTC ATA GCC TGA AGA ACG -3′.

To generate *Mecp2*/*Nfkb1* double mutant mice for Golgi analysis and Kaplan–Meier survival analysis, female *Mecp2*^+/−^ mice were first crossed with male *Nfkb1*^−/−^ mice, and the progeny were then interbred. Kaplan–Meier survival curves were generated, and log-rank tests were performed using GraphPad Prism 5 (GraphPad Software, La Jolla, CA).

### CPN labelling, dissociation and purification

CPN were retrogradely labelled with green fluorescent microspheres (Lumafluor Corp., FL) by injection into contralateral cortex. In brief, green fluorescent microspheres were stereotaxically injected into the CPN axon terminal field in one hemisphere in male wild-type or *Mecp2*-null P3 mice with a digitally controlled oocyte injector (Drummond, Broomall, PA), using pulled glass micropipets with tip diameter of 30–60 μm. Green fluorescent microspheres were retrogradely transported across the corpus callosum to the contralateral hemisphere. At P14, mice were deeply anaesthetized, and the regions of labelled sensorimotor cortex were microdissected. Labelled cortices were dissected in cold dissociation medium (20 mM glucose, 0.8 mM kynurenic acid, 0.05 mM DL-2-amino-5-phosphonopentanoic acid (APV), 50 U ml^−1^ penicillin–0.05 mg ml^−1^ streptomycin, 0.09 M Na_2_SO_4_, 0.03 M K_2_SO_4_ and 0.014 M MgCl_2_), and enzymatically digested in dissociation medium containing 0.32 mg l^−1^ L-cysteine HCl and 20 U ml^−1^ papain (Worthington, Lakewood, NJ) at 37 °C for 45 min, followed by rinsing with OptiMem (4 °C) (Life Technologies, Gaithersburg, MD) containing 20 mM glucose, 0.4 mM kynurenic acid and 0.025 mM APV. Cortices were mechanically triturated using fire-polished Pasteur glass pipets to create a single-cell suspension, and were pooled in RNA*later*. All chemicals were purchased from Sigma-Aldrich (St Louis, MO), unless stated otherwise. We collected three independent sets of *Mecp2*-null and wild-type P14 CPN for microarray analysis, and another three sets for qPCR experiments. For each of three biological replicates for each sample type, approximately 20,000 CPN were purified from pooled dissociated cells (from approximately 10 cortices), using a BD FACS Vantage SE DiVa cell sorter. Cells were gated based on green fluorescence, and forward and side scatter gates were set to select the population of large projection neurons.

### Affymetrix microarray analysis

RNA was extracted from FACS-purified CPN using the StrataPrep Total RNA Micro Kit (Stratagene, La Jolla, CA), and amplified according to the Affymetrix small sample protocol, using two consecutive rounds of linear *in vitro* transcription. To ensure reproducibility and biological significance, independent RNA samples were collected from three independent FACS purifications for both *Mecp2*-null and wild-type CPN. We performed microarray analysis using Affymetrix M430 2.0 GeneChips (Affymetrix, Santa Clara, CA), which include probe sets for over 39,000 mouse genes and ESTs. Data were analysed using Rosetta Resolver analysis software (Rosetta Biosoftware, Seattle, WA). All microarray data have been deposited in the Gene Expression Omnibus database at NCBI (Accession GSE50225).

### Quantitative real-time PCR (qPCR)

RNA was extracted using TRIzol (Invitrogen, Carlsbad, CA), and cDNA was synthesized using SuperScriptII reverse transcriptase (Invitrogen). qPCR was performed with a LightCycler 1.5 system (Roche, Branford, CT) according to the manufacturer's instructions. Primer pairs for *Irak1*, *Camk2d*, *Tnf*, *Mecp2* and *Gapdh* were as follows; each primer of each primer pair was designed in different exons, so as not to amplify genomic DNA:

*Irak1*: Forward 5′- ACTACATATGCTGTGAAGAGA -3′

   Reverse 5′- CTCATCCAGAAGCACGTTAGA -3′

*Camk2d*: Forward 5′- CACCGACGAGTATCAGCTCTT -3′

    Reverse 5′- CCACTATGTCTTCAAACAGTT -3′

*Tnf*: Forward 5′- ACCATGAGCACAGAAAGCATG -3′

 Reverse 5′- AGAAGATGATCTGAGTGTGAG -3′

*Mecp2*: Forward 5′- GCCGATCTGCTGGAAAGTAT -3′

   Reverse 5′- CCTCTCCCAGTTACCGTGAA -3′

*Gapdh*: Forward 5′- GGCATTGCTCTCAATGACAA -3′

   Reverse 5′- TGTGAGGGAGATGCTCAG TG -3′

Each PCR reaction consisted of 1X LightCycler FastStart DNA Master SYBR Green I mixture, 0.125–0.25 μM primers, and cDNA. We generated a standard curve for each gene, and performed relative quantification analysis in triplicate for each sample, using three independent RNA samples from each genotype. The results are reported as the ratio of target DNA sequence to a calibrator sample, following normalization to a reference gene, *Gapdh*. The average of the ratios of wild-type samples were set as 1. For experiments using FACS-purified CPN samples, the data were analysed using the paired two-sided *t*-test for each pair of pooled wild-type and *Mecp2*-null CPN, which were sorted by FACS at the same time. For all other qPCR experiments, the data were analysed with an unpaired two-sided t-test. To verify the specificity of the amplicons, we ran the amplicons on agarose gels and confirmed the molecular size of the amplicons, in addition to melting curve analysis. Error bars indicate s.e.m.

### Immunocytochemistry

Immunocytochemistry was performed following standard protocols. Briefly, male postnatal pups were transcardially perfused with phosphate-buffered saline (PBS), then with 4% PFA, dissected, and post-fixed in 4% paraformaldehyde overnight. Brains were sectioned at 50 μm on a vibrating microtome (Leica). Floating sections were subjected to antigen retrieval in 95 degree 0.01 M citric acid pH 6.0 for 10 min, blocked with 4% goat or donkey serum, 0.3% BSA, 0.3% Triton X-100 (Sigma-Aldrich) and 0.025% Sodium Azide in PBS for 30 min. Primary antibodies were diluted in appropriate blocking solution, and incubated with sections overnight. The following day, sections were rinsed three times with PBS, and incubated with appropriate secondary antibodies diluted in blocking solution for 3 h at room temperature. Sections were again rinsed three times with PBS, and mounted using Fluoromount (SouthernBiotech) for image acquisition.

Antibody dilutions were as follows: rabbit α-Irak1 (1:200, Abcam ab238); rabbit α–MeCP2 (1:500, Cell Signaling Ab#3456); rabbit α−NF-κB P65 (1:200, Cell Signaling Ab#8242); mouse α-NeuN (1:500, Chemicon MAB377); goat α-vesicular acetylcholine transporter (1:500, Millipore ABN100); rat α-somatostatin (1:200, Millipore MAB354); and chick anti-GFP IgG antibody (1:1500, Millipore AB16901). All antibodies are commercially available, and validated. All immunocytochemistry experiments were performed on a minimum of three animals and/or independent cultures; representative images are shown. The expression pattern of IRAK1 was confirmed with an independent antibody (Cell Signaling, Ab#4504). For consistency, all images depicted are of the Abcam antibody. Appropriate secondary antibodies from the Molecular Probes Alexa Series were used (1:500, Invitrogen).

Images were acquired using a Nikon E90i microscope with a 1.5 megapixel cooled CCD digital camera (Andor Technology), and Elements acquisition software (Nikon Instruments). For confocal imaging (Fig. 1e,f; Figs 3b and 4a), a Zeiss LSM 780 confocal microscope was used with Zen acquisition software (Zeiss).

### Western blots

Cortex and heart tissues were homogenized, protein extracted, and immunoblotting performed following standard protocols. Total protein was quantified using BCA Protein Assay Kit (Thermo Scientific), as per manufacturer's instructions, and 50 μg total protein was loaded per sample. The following primary antibodies were employed: rabbit α−NF-κB P65 (1:1,000, Cell Signaling Ab#8242) and rabbit α−Phospho-NF-κB P65 Ser536 (1:1,000; Cell Signaling Ab#3033); mouse α-β-actin (1:2,000; Sigma A5441). Appropriate horseradish peroxidase (HRP)-conjugated secondary antibodies were from Pierce, and signal was detected with SuperSignal West Pico Chemiluminescent Substrate (Pierce), and a FluorChem M imager (ProteinSimple). Even loading was confirmed by β-actin, and densitometry was performed on total p65 and phospho-p65 bands using ImageJ (http://rsb.info.nih.gov/ij/index.html) from three pairs of wild-type and three *Mecp2*-null male littermates. Images have been cropped for presentation. Full-size images are presented in Supplementary Fig. 5. Data were assessed by unpaired two-sided *t*-test. Error bars indicate s.e.m.

### Plasmids

To overexpress *Irak1* in neural precursor cultures, *Irak1* (NCBI Accession BC004778) was subcloned into a pCBIG vector containing IRES-EGFP under the control of a constitutively active CMV/β-actin promoter (generous gift of C. Lois, MIT). To overexpress *Irak1* in post-mitotic differentiated neurons *in vivo*, we constructed a pTIG vector by replacing the CMV/β-actin promoter of the pCBIG vector with a mouse *tau* promoter (generous gift of A. Andreadis, Shriver Center for Mental Retardation)^69^ and *Irak1* was subcloned downstream of the promoter. To knock down *Mecp2* expression, a construct consisting of a bicistronic cassette encoding an shRNA sequence (GTCAGAAGACCAGGATCTC ) targeted against *Mecp2* driven by a U6 promoter, and GFP driven by an ubiquitin, promoter was used. In control experiments, a scrambled sequence (AGTAACCTGACGGAGTACC ) replaced the *Mecp2* shRNA (both constructs were a generous gift of Dr. Z. Zhou, University of Pennsylvania^29^). To measure NF-κB activation, a plasmid containing five copies of an NF-κB response element driving expression of the luciferase reporter gene luc2P was purchased from Promega (Cat# E8491). Relative luminescence was normalized to a co-transfected Renilla luciferase construct, derived from the psiCHECK-2 vector (Promega, Cat# C8021) with the HSV-TK promotor and Firefly luciferase cut out by digestion with Not1 and Xba1.

### Embryonic CNS neural precursor culture

Timed pregnant E13.5 female CD1 mice were deeply anaesthetised with Avertin to obtain E13.5 mouse embryos. Embryos (male and female) were dissected in Hank's buffered saline solution (HBSS), neocortical tissue was dissociated by gentle mechanical trituration in HBSS, and cells were collected by centrifugation.

Dissociated cells were resuspended in DMEM/F12-based serum-free growth medium containing N2 supplement, B27 supplement (Invitrogen) and FGF2 (20 ng ml^−1^) (PeproTech, Rocky Hill, NJ), and plated on glass coverslips precoated with poly-L-ornithine (50 μg ml^−1^) and fibronectin (1 μg ml^−1^) (Sigma-Aldrich) at 40,000 cells cm^−2^. After 2 days in proliferation medium, neural precursors were treated with differentiation media (proliferation medium with 2% fetal bovine serum (Invitrogen), but without FGF2). After 3 days in differentiation medium, differentiated cells were transfected with pCBIG or pCBIG-Irak1 plasmids containing EGFP as a reporter, using Lipofectamine LTX and Plus Reagent (Invitrogen). After 2 days, cells were fixed in 4% paraformaldehyde in PBS, permeabilized with 0.1% Triton X100, preincubated with PBS containing 10% goat serum and then incubated with primary antibodies overnight at 4 °C. Primary antibodies were used at the following concentrations: chicken polyclonal anti-GFP antibody (1:1,000 Millipore AB16901); mouse monoclonal anti-MAP2 antibody (1:500, Millipore M1406); mouse α–Tuj1 (1:500, βIII tubulin, Covance MMS-435 P); rabbit α-Irak1 (1:200, Abcam ab238); rabbit α–MeCP2 (1:500, Cell Signaling Ab#3456); and rabbit α−NF-κB P65 (1:200, Cell Signaling Ab#8242) Cells were washed with PBS, and incubated in fluorescent secondary antibodies for 2 h at room temperature. Appropriate secondary antibodies from the Molecular Probes Alexa Series were used (1:500, Invitrogen). We traced the dendrites of transfected neurons positive for both GFP and MAP2 (*n*=16 for pCBIG; *n*=20 for pCBIG-Irak1; from four independent coverslips for each condition). For Sholl analysis, concentric circles in 20 μm radius increments were superimposed around the centre of the soma, and the number of dendrites crossing each circle were quantified under blind conditions. Data were assessed by unpaired two-sided *t*-test. Error bars indicate s.e.m.

### *In vivo* overexpression of *Irak1*

Timed pregnant CD1 mice with E15.5 embryos were anaesthetised, and an incision was made in the abdomen, exposing the uterine horns. One microgram of plasmid DNA (1.0 μg μl^−1^) mixed with 0.005% Fast Green in sterile PBS was injected *in utero* into the lateral ventricle of E15.5 embryos (male and female), using pulled glass micropipets with tip diameter of 30–60 μm, beveled at 15°. Electroporation of the plasmids was performed by placing a positive electrode above the cortex and a negative electrode behind the head, and applying five pulses of current at 30 V for 50 ms per pulse with 1-s intervals between pulses using a CUY21EDIT square wave electroporator (Nepa Gene, Japan). This introduced the construct into precursors lining the ventricle. At P14, electroporated pups were perfused, their brains were fixed in 4% PFA overnight, and brains were sectioned at a thickness of 100 μm with a vibrating microtome (Leica, Bannockburn, IL). GFP-positive electroporated layer 2/3 pyramidal neurons were systematically selected for Sholl analysis with the experimenter blind to the condition (*n*=24 from three independent electroporated brains for pTIG; *n*=28 from four independent electroporated brains for pTIG-Irak1). Images were captured, and image series were reconstructed using Photoshop (Adobe, San Jose, CA). Sholl analysis was performed as described above.

### *In vivo* knockdown of *Mecp2*

One μg of either the control scrambled or *Mecp2* shRNA plasmid (1.0 μg μl^−1^) mixed with 0.005% Fast Green in sterile PBS was injected *in utero* into the lateral ventricle of E15.5 CD1 embryos, and *in vivo* electroporations were performed as described for the overexpression of *Irak1*. At P14, mice were deeply anesthetized, and the regions of GFP-labelled sensorimotor cortex were microdissected and dissociated as described for the retrogradely labelled CPN. We collected three independent sets of *Mecp2* shRNA and control scrambled shRNA electroporated cells. For each of three biological replicates for each sample type, approximately 100,000–200,000 cells were purified from pooled dissociated cells from 3–4 labelled cortices, using a BD FACS Vantage SE DiVa cell sorter. Cells were gated based on green fluorescence, and forward and side scatter gates were set to select the population of large projection neurons. RNA was extracted from FACS-purified cells using the StrataPrep Total RNA Micro Kit (Agilent Technologies), and cDNA was synthesized using SuperScript II reverse transcriptase (Invitrogen). qPCR was performed as described for the *Mecp2*-null and wild-type samples, using the same *Irak1* and *Gapdh* primers. The results are reported as the ratio of target DNA sequence to a calibrator sample, following normalization to *Gapdh*. The average of the scrambled shRNA samples was set at 1. The data were analysed using the paired two-sided *t*-test for each pair of pooled *Mecp2* shRNA and scrambled shRNA cells, which were sorted by FACS at the same time.

### NF-κB luciferase reporter assays

P1 C57Bl/6 wild-type brains (male and female) were dissected and dissociated as described for E13.5 cortical cultures. Dissociated cells were nucleofected with the NF-κB reporter construct and control Renilla luciferase construct, along with one of the following: scrambled shRNA, *Mecp2* shRNA or *Irak1* expression construct, using an Amaxa Mouse Neuron Nucleofector kit (Lonza), and the Amaxa Nucleofector II Device (Lonza). P1 Mecp2−/y and Mecp2+/y brains (male) were prepared as described above, and nucleofected with the NF-κB reporter construct and control Renilla luciferase construct. Cells were cultured for 48 h at high density in 96-well plates coated with poly-D-lysine (Sigma-Aldrich), in growth media composed of 50% DMEM-F12 and 50% Neurobasal (Gibco), with N2, B27 and GlutaMax supplements (Invitrogen). For vitamin D experiments, 0, 10 or 100 μM 1α2,5-dihydroxyvitamin D3 (Sigma-Aldrich) was added to the culture media at 24 h. 1α2,5-dihydroxyvitamin D3 is reconstituted in ethanol, and the same small volume of ethanol was added to cultures at each vitamin D concentration. At 48 h, Firefly and Renilla luciferase activities were measured using the Dual-Glo Luciferase Assay system (Promega) and a GloMax 96 microplate luminometer (Promega). The luminescence of each well was normalized individually, and triplicate wells were averaged within each experiment. Relative luminescence was normalized to the control, shScram experimental condition, and data represent four independent biological replicates. Data were assessed by unpaired two-sided t-test. Error bars indicate s.e.m.

### Golgi staining

Golgi staining was carried out using a rapid Golgi method. Briefly, male brains at 8 weeks of age were incubated in a solution containing 2% potassium dichromate and 0.2% osmium tetroxide for 7 days in the dark. Brains were then rinsed with 0.5% silver nitrate until precipitate disappeared, and were incubated in 1% silver nitrate in the dark for 5 days at room temperature. Brains were dehydrated and embedded in low-viscosity nitrocellulose. The brains were cut at a thickness of 100 μm, and cleared in α-terpineol. Layer 2/3 pyramidal neurons were systematically selected with the experimenter blind to the condition, and were drawn using a camera lucida device (*Mecp2*^+/y^;*Nfkb1*^+/+^, *n*=30 from three brains; *Mecp2*^+/y^;*Nfkb1*^−/−^, *n*=18 from three brains; *Mecp2*^−/y^;*Nfkb1*^+/+^, *n*=18 from two brains; *Mecp2*^−/y^;*Nfkb1+/−*, *n*=24 from three brains; *Mecp2*^−/y^; and *Nfkb1*^−/−^, *n*=20 from two brains). The reconstructed neuronal drawings were analysed for the number of primary and secondary dendrites, and branch points. Data were assessed by unpaired two-sided *t*-test. Error bars indicate s.e.m.

### Statistical methods

No statistical methods were used to pre-determine sample sizes, but our sample sizes are similar to those generally employed in the field. We used a two-way analysis of variance (ANOVA) and the Bonferroni test for Sholl analysis, and a two-sided *t*-test procedure to determine statistical significance for other analyses. Data distribution was handled as if normal, but this was not formally tested (since potential differences in results would be minor). Variance between groups was analysed using the f-test procedure. Only for the survival curve analysis, we used the log-rank test, as this method is commonly used to compare the survival distributions of two groups.

## Additional information

**Accession codes:** All microarray data have been deposited in the Gene Expression Omnibus database at NCBI (Accession GSE50225).

**How to cite this article:** Kishi, N. *et al*. Reduction of aberrant NF-κB signaling ameliorates Rett syndrome phenotypes in *Mecp2*-null mice. *Nat. Commun.* 7:10520 doi: 10.1038/ncomms10520 (2016).

## Supplementary Material

Supplementary Information Supplementary Figures 1-5, Supplementary Tables 1-5 and Supplementary Methods
