# Supplementary material for: Reduction of aberrant NF-κB signalling ameliorates Rett syndrome phenotypes in Mecp2-null mice
Source: Nat Commun. 2016 Jan 29;7:10520. doi: 10.1038/ncomms10520 (PMC4740176; doi:10.1038/ncomms10520)
Supplement: Supplementary Information — Supplementary Figures 1-5, Supplementary Tables 1-5 and Supplementary Methods [file ncomms10520-s1.pdf]

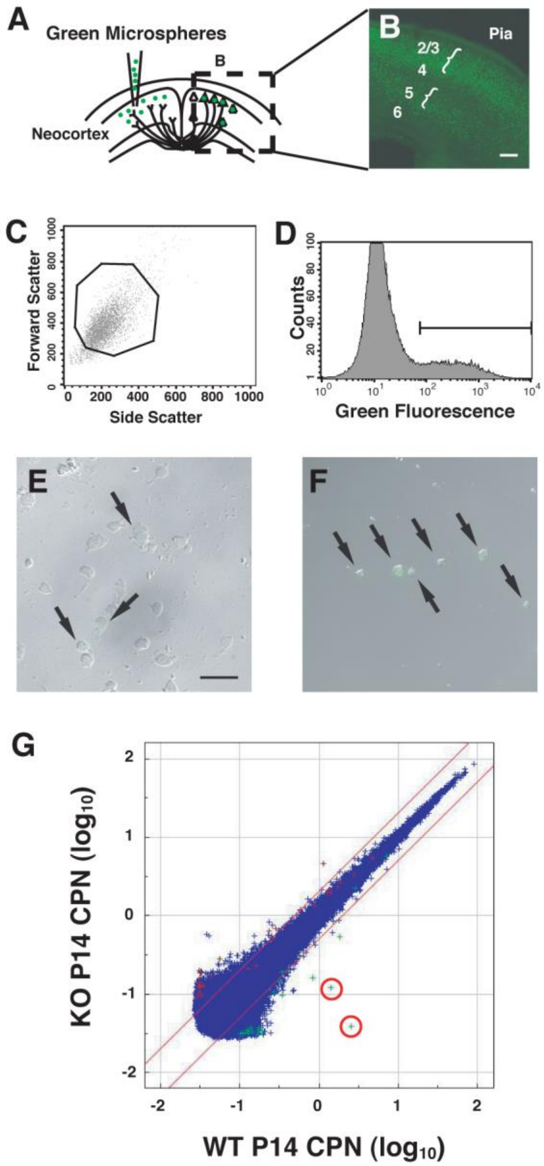

Supplementary Figure 1: Identification of dysregulated genes in *Mecp2*-null FACS-purified CPN
(A-B) At P3, green fluorescent microspheres were injected in one hemisphere of the neocortex of wild-type and *Mecp2*-null mice in CPN axonal terminal fields, and were retrogradely transported to the somata of contralateral CPN. The brackets in B indicate retrogradely labeled layer 2/3 and 5 CPN. (C-D) FACS plot of the population of purified CPN; CPN were selected based on (C) size (forward scatter) and surface characteristics (side scatter), and (D) high intensity green fluorescence. (E-F) Dissociated P14 cells before (E) and after (F) FACS. Arrows in E and F indicate CPN identified by green fluorescent microspheres. (G) Intensity plots of all genes in pair-wise comparisons of wild-type vs. *Mecp2*-null CPN at P14. We performed microarray analysis on three sets of independent biological replicates, using Affymetrix M430 2.0 GeneChips, which include probe sets for over 39,000 mouse genes and ESTs; differentially expressed genes (p-value < 0.001) indicated by red crosses (over-expressed) and green crosses (under-expressed). Red lines mark the 2-fold change interval. Red circles indicate *Mecp2* probe sets. Scale bar 200 μm in B, 50 μm in E and F.

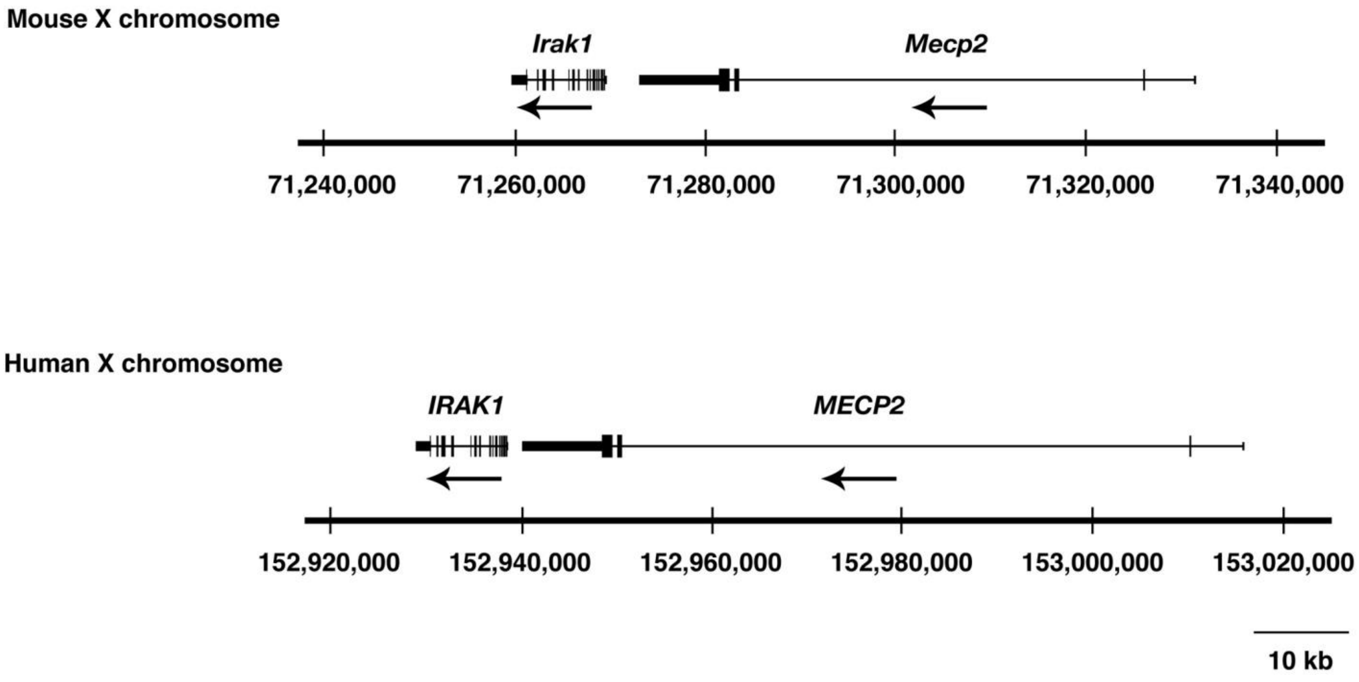

Supplementary Figure 2: Location of *Irak1*/*IRAK1* and *Mecp2*/*MECP2* on mouse and human X chromosome
The *Irak1*/*IRAK1* locus is located downstream of the *Mecp2*/*MECP2* locus on both the mouse and human X chromosome. Positions on the X chromosome are based on the NCBI Build 37 assembly for the mouse genome, and the NCBI Build 36.1 assembly for the human genome.

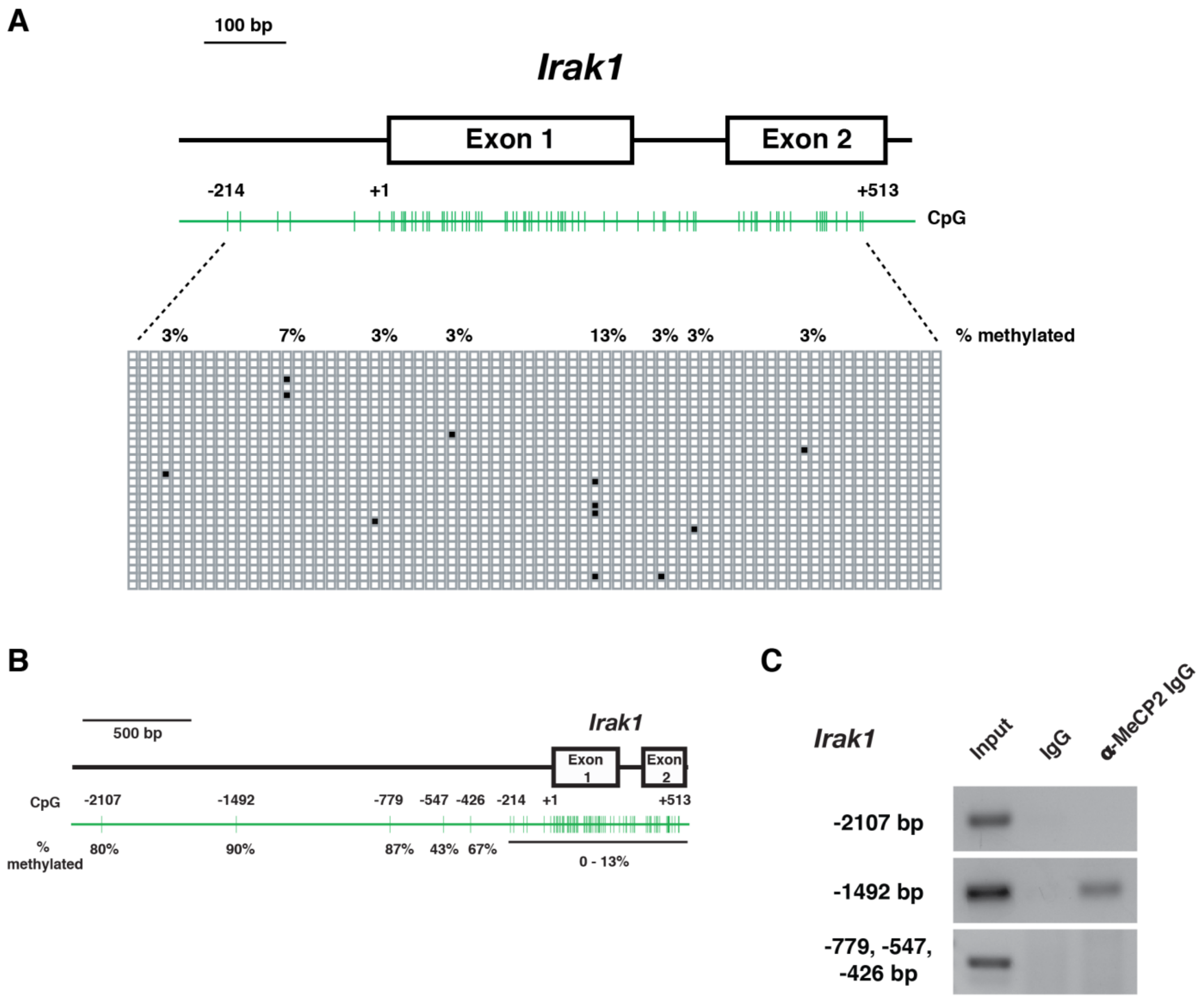

Supplementary Figure 3: Methylation status of CpGs in exons 1 and 2 of *Irak1*
(A) Methylation status of 74 CpGs near the transcription initiation site of *Irak1* in the cortex of P14 male mice was analyzed by bisulfite genomic sequencing. In contrast to the 5 CpGs (-2107, -1492, -779, -547, and -426 bp) on the *Irak1* promoter region, these CpGs were methylated at very low levels. Closed boxes, methyl-CpG; open boxes, unmethyl-CpG. Locations of CpG sites in the *Irak1* promoter region are relative to the transcription initiation site. (B) While most of the CpGs on or near exons 1 and 2 of *Irak1* are methylated at very low levels in P14 cortex, 5 CpGs upstream of the TIS of *Irak1* (-2107, -1492, -779, -547, and -426 bp) are highly methylated. Closed boxes, methylated-CpG; open boxes, unmethylated-CpG. (C) ChIP with anti-MeCP2 IgG identifies that MeCP2 specifically binds to the CpG located 1492 bp upstream of the TIS of *Irak1*, and not to the other 4 highly methylated CpGs. There is no binding with control IgG to any of these sites.

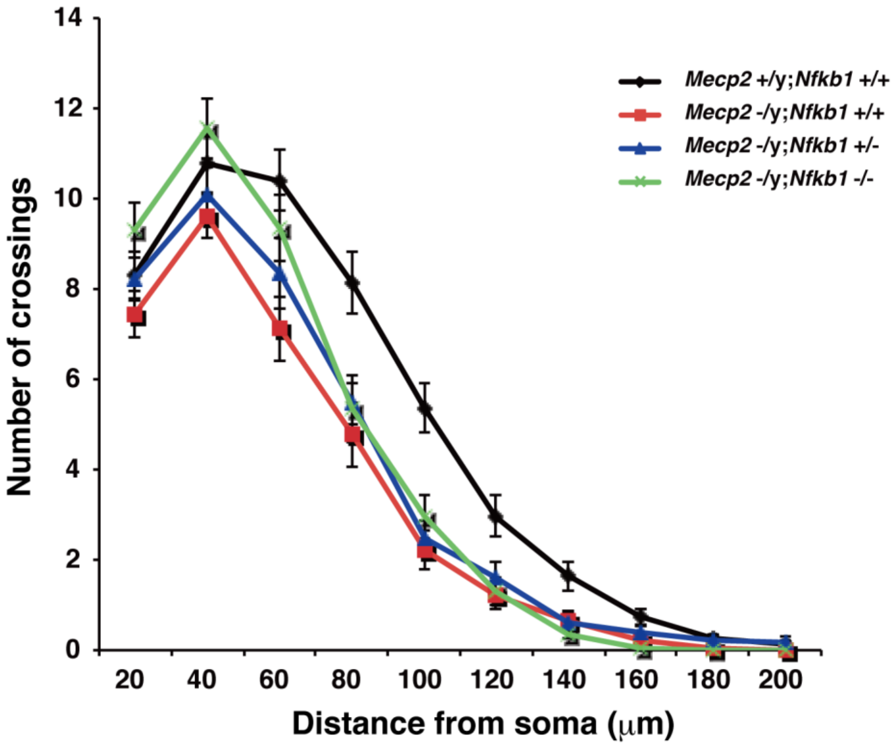

Supplementary Figure 4: Sholl analysis does not detect improvement of *Mecp2*-null dendritic complexity by attenuated NF-κB signaling

To investigate whether modification of NF-κB signaling rescue dendritic complexity of *Mecp2*-null layer 2/3 pyramidal neurons, we crossed *Mecp2* mutant mice with *Nfkb1* mutant mice. We visualized the dendritic morphology of *Mecp2*$^{+/y}$;*Nfkb1*$^{+/+}$ (black line), *Mecp2*$^{-/y}$;*Nfkb1*$^{+/+}$ (red line), *Mecp2*$^{-/y}$;*Nfkb1*$^{+/-}$ (blue line), and *Mecp2*$^{-/y}$;*Nfkb1*$^{-/-}$ (green line) layer 2/3 pyramidal neurons by Golgi staining. Although we analyzed an initial (relatively low sensitivity) screen of dendritic complexity by Sholl analysis, we failed to detect significant differences in *Mecp2*-null dendritic complexity with additional disruption of *Nfkb1*.

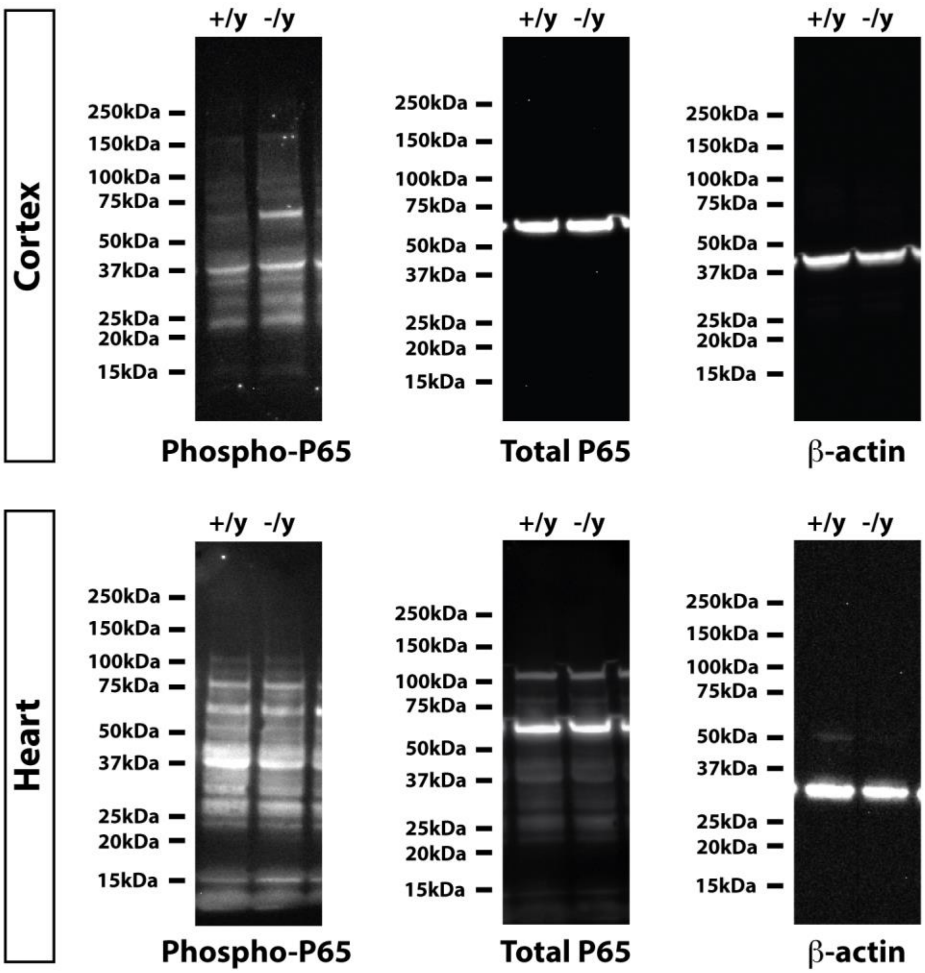

Supplementary Figure 5: Phosphorylated P65 is increased relative to total P65 in cortex of Mecp2-nulls
We investigated NF-kB activation in vivo in 8 week neocortex and heart by Western blot for phosphorylated p65/RelA, relative to total P65/RelA, and normalized to B-actin loading control. We identified an approximately 2-fold up-regulation of phospho-P65 in Mecp2-/y cortex compared to littermates controls, but there was no significant difference in heart. Blots display representative data from one pair of littermates. Quantification was performed on three independent pairs of littermates. These images have been cropped for presentation in Figure 3E.

Supplementary Table 1: Genes over-expressed by *Mecp2*-null P14 CPN (fold change > 2.0, *P* < 0.001)

Gene Symbol	NCBI Accession	Description	Fold Change *Mecp2*-null vs. WT	*P* Value
Rab39b	AK020665	RAB39B, member RAS oncogene family	3.90	4.53E-08
Kif1b	BE199508	Kif1b kinesin family member 1B	6.25	2.91E-07
Irak1	AV091354	interleukin-1 receptor-associated kinase 1	2.80	2.09E-06
Plekha1	BB475301	Pleckstrin homology domain containing, family A (phosphoinositide binding specific) member 1	5.28	2.00E-05
Cpm	BB549552	carboxypeptidase M	7.83	3.00E-05
Cst12	AF440737	cystatin 12	3.97	6.00E-05
	BB102018	BB102018, EST	2.25	1.70E-04
Igh-VJ558	AK007826	immunoglobulin heavy chain (J558 family)	4.50	2.30E-04
5830434F19Rik	AK017969	RIKEN cDNA 5830434F19 gene	5.32	2.90E-04
Utp23	AK005831	rRNA-processing protein UTP23 homolog	5.69	4.50E-04
Tas2r119	NM_020503	taste receptor, type 2, member 119	4.71	4.70E-04
1700082M22Rik	NM_028564	RIKEN cDNA 1700082M22 gene	3.98	6.70E-04
Gpatch8	BC019948	G patch domain containing 8	2.03	6.70E-04
Klra12	AF204265	killer cell lectin-like receptor subfamily A, member 12	3.09	7.90E-04
Ptprk	BB667296	protein tyrosine phosphatase, receptor type, K	6.10	8.00E-04
2300010F08Rik	AK009054	RIKEN cDNA 2300010F08 gene	4.12	9.30E-04
Dep1	BB626684	diabetic embryopathy 1	3.71	9.80E-04
V1rd1	NM_030742	vomeronasal 1 receptor, D1	3.02	9.90E-04

Supplementary Table 2: Genes under-expressed by *Mecp2*-null P14 CPN (fold change < -2.0, *P* < 0.001)

Gene Symbol	NCBI Accession	Description	Fold Change *Mecp2*-null vs. WT	*P* Value
Mecp2	BB130002	methyl CpG binding protein 2	-83.78	0.00E+00
Mecp2	BC027153	methyl CpG binding protein 2	-12.71	1.28E-16
Cd99l2	BB334959	CD99 antigen-like 2	-3.43	1.36E-06
Cd99l2	BB334959	CD99 antigen-like 2	-5.79	4.33E-06
Ankrd17	BG065567	Ankyrin repeat domain 17	-3.66	6.39E-06
Pde4b	BG793493	phosphodiesterase 4B, cAMP specific	-4.77	2.00E-05
A630023P12Rik	AI451630	RIKEN cDNA A630023P12 gene	-5.26	7.00E-05
Igbp1b	AK006203	immunoglobulin (CD79A) binding protein 1b	-5.67	2.10E-04
Gsn	NM_010354	gelsolin	-3.65	2.50E-04
Gad1	AF326547	glutamic acid decarboxylase 1	-2.84	2.80E-04
	AW912607	uf54a03.x1, EST	-3.82	3.10E-04
	BQ031094	UI-1-CF0-alz-c-03-0-UI.s1, EST	-2.14	4.50E-04
Nfatc1	BB356861	nuclear factor of activated T-cells, cytoplasmic, calcineurin-dependent 1	-4.60	4.60E-04
D3Ertd300e	NM_019995	DNA segment, Chr 3, ERATO Doi 300, expressed	-6.54	4.70E-04
C330006K01Rik	AV263612	RIKEN cDNA C330006K01 gene	-4.92	4.80E-04
Hsd17b13	AV050442	hydroxysteroid (17-beta) dehydrogenase 13	-4.13	5.10E-04
Thumpd2	AK012806	THUMP domain containing 2	-4.11	5.80E-04
Mcf2	NM_133197	mcf.2 transforming sequence	-3.99	6.10E-04
	BB830387	BB830387, EST	-2.46	6.60E-04
	BB341238	BB341238, EST	-5.42	9.10E-04
2210010C04Rik	AK008695	RIKEN cDNA 2210010C04 gene	-5.22	9.50E-04

Supplementary Table 3: Genes over-expressed by *Mecp2*-null P14 CPN (fold change > 1.5, *P* < 0.005)

Gene Symbol	NCBI Accession	Description	Fold Change *Mecp2*-null vs. WT	*P* Value
Rab39b	AK020665	RAB39B, member RAS oncogene family	3.90	4.53E-08
Nhsl2	XM_00147828	NHS-like 2	1.58	1.78E-07
Kif1b	BE199508	Kif1b kinesin family member 1B	6.25	2.91E-07
Irak1	AV091354	interleukin-1 receptor-associated kinase 1	2.80	2.09E-06
Plekha1	BB475301	Pleckstrin homology domain containing, family A (phosphoinositide binding specific) member 1	5.28	2.00E-05
Fbxw10	NM_001033669	F-box and WD-40 domain protein 10	1.78	2.00E-05
Cpm	BB549552	carboxypeptidase M	7.83	3.00E-05
Cst12	AF440737	cystatin 12	3.97	6.00E-05
Usp6nl	NM_001080548	USP6 N-terminal like	1.51	1.20E-04
	BB102018	BB102018, EST	2.25	1.70E-04
Igh-VJ558	AK007826	immunoglobulin heavy chain (J558 family)	4.50	2.30E-04
5830434F19Rik	AK017969	RIKEN cDNA 5830434F19 gene	5.32	2.90E-04
Utp23	AK005831	rRNA-processing protein UTP23 homolog	5.69	4.50E-04
Pcdh17	NM_001013753	protocadherin 17	1.76	4.60E-04
Tas2r119	NM_020503	taste receptor, type 2, member 119	4.71	4.70E-04
1700082M22Rik	NM_028564	RIKEN cDNA 1700082M22 gene	3.98	6.70E-04
Gpatch8	BC019948	G patch domain containing 8	2.03	6.70E-04
Klra12	AF204265	killer cell lectin-like receptor subfamily A, member 12	3.09	7.90E-04
Ptprk	BB667296	protein tyrosine phosphatase, receptor type, K	6.10	8.00E-04
2300010F08Rik	AK009054	RIKEN cDNA 2300010F08 gene	4.12	9.30E-04
Dep1	BB626684	diabetic embryopathy 1	3.71	9.80E-04
V1rd1	NM_030742	vomeronasal 1 receptor, D1	3.02	9.90E-04
Il18bp	NM_010531	Il18bp interleukin 18 binding protein	4.04	1.16E-03
Irak1	NM_008363	interleukin-1 receptor-associated kinase 1	2.79	1.24E-03
Naaladl2	XM_91083	N-acetylated alpha-linked acidic dipeptidase-like 2	3.83	1.29E-03

Gene Symbol	NCBI Accession	Description	Fold Change *Mecp2*-null vs. WT	*P* Value
	BM249512	BM249512, EST	3.98	1.37E-03
Unc5b	NM_029770	Unc5b unc-5 homolog B (C. elegans)	4.80	1.59E-03
Tmem2	NM_001033759	transmembrane protein 2	1.79	1.61E-03
Gpr107	NM_178760	G protein-coupled receptor 107	1.66	1.63E-03
Efcab3	NM_001081046	EF-hand calcium binding domain 3	3.03	1.67E-03
Ttll10	NM_029264	tubulin tyrosine ligase-like family, member 10	2.44	1.70E-03
Atp2c1	NM_175025	ATPase, Ca++-sequestering	2.85	1.96E-03
Mei1	XM_901649	Mei1 meiosis defective 1	4.08	1.97E-03
A630038E17Rik	BC038285	RIKEN cDNA A630038E17 gene	3.91	2.03E-03
Irak1	NM_008363	interleukin-1 receptor-associated kinase 1	3.17	2.09E-03
Nrip1	NM_173440	nuclear receptor interacting protein 1	1.97	2.23E-03
LOC552912	XR_005134	hypothetical LOC552912	1.54	2.36E-03
Camk2d	NM_001025438	calcium/calmodulin-dependent protein kinase II, delta	2.00	2.40E-03
Wdfy3	XM_152907	WD repeat and FYVE domain containing 3	2.05	2.60E-03
	AK045858	AK045858, EST	2.53	2.64E-03
Klk5	NM_008456	Klk1b5 kallikrein 1-related peptidase b5	4.44	2.86E-03
	BE995622	UI-M-CG0p-bia-e-07-0-UI.s1, EST	3.47	2.89E-03
4933439C20Rik	NR_003518	RIKEN cDNA 4933439C20 gene	1.64	3.39E-03
Lpin2	NM_022882	Lipin 2	4.01	3.49E-03
4933402E13Rik	XM_889482	RIKEN cDNA 4933402E13 gene	2.89	3.74E-03
5033405D04Rik	AK019887	RIKEN cDNA 5033405D04 gene	2.38	3.85E-03
Neurl3	NM_153408	Neurl3 neuralized homolog 3 homolog (Drosophila)	4.92	3.94E-03
Obox5	NM_145709	oocyte specific homeobox 5	2.71	4.04E-03
Grip1	AK086934	Glutamate receptor interacting protein 1	4.15	4.13E-03
D18Ertd653e	NM_172631	DNA segment, Chr 18, ERATO Doi 653, expressed	3.56	4.31E-03
Ovgp1	NM_007696	oviductal glycoprotein 1	2.36	4.47E-03
Casr	NM_013803	calcium-sensing receptor	3.95	4.49E-03
Rab34	NM_033475	RAB34, member of RAS oncogene family	1.84	4.68E-03

Gene Symbol	NCBI Accession	Description	Fold Change *Mecp2*-null vs. WT	*P* Value
	AK084017	AK084017, EST	1.60	4.69E-03
Adora2a	NM_009630	adenosine A2a receptor	2.18	4.82E-03
Tcfe2a	NM_011548	transcription factor E2a	5.06	4.92E-03

Supplementary Table 4: Genes under-expressed by *Mecp2*-null P14 CPN (fold change < -1.5, *P* < 0.005)

Gene Symbol	NCBI Accession	Description	Fold Change *Mecp2*-null vs. WT	*P* Value
Mecp2	BB130002	methyl CpG binding protein 2	-83.78	0.00E+00
Mecp2	BC027153	methyl CpG binding protein 2	-12.71	1.28E-16
Cd99l2	BB334959	CD99 antigen-like 2	-3.43	1.36E-06
Cd99l2	BB334959	CD99 antigen-like 2	-5.79	4.33E-06
Ankrd17	BG065567	Ankyrin repeat domain 17	-3.66	6.39E-06
Pde4b	BG793493	phosphodiesterase 4B, cAMP specific	-4.77	2.00E-05
A630023P12Rik	AI451630	RIKEN cDNA A630023P12 gene	-5.26	7.00E-05
Mycn	NM_008709	v-myc myelocytomatosis viral related oncogene	-1.93	1.40E-04
Igbp1b	AK006203	immunoglobulin (CD79A) binding protein 1b	-5.67	2.10E-04
Ccdc126	NM_175098	coiled-coil domain containing 126	-1.90	2.40E-04
Gsn	NM_010354	gelsolin	-3.65	2.50E-04
Gad1	AF326547	glutamic acid decarboxylase 1	-2.84	2.80E-04
	AW912607	uf54a03.x1, EST	-3.82	3.10E-04
	BQ031094	UI-1-CF0-alz-c-03-0-UI.s1, EST	-2.14	4.50E-04
Nfatc1	BB356861	nuclear factor of activated T-cells, cytoplasmic, calcineurin-dependent 1	-4.60	4.60E-04
D3Ertd300e	NM_019995	DNA segment, Chr 3, ERATO Doi 300, expressed	-6.54	4.70E-04
C330006K01Rik	AV263612	RIKEN cDNA C330006K01 gene	-4.92	4.80E-04
Hsd17b13	AV050442	hydroxysteroid (17-beta) dehydrogenase 13	-4.13	5.10E-04
Thumpd2	AK012806	THUMP domain containing 2	-4.11	5.80E-04
Mcf2	NM_133197	mcf.2 transforming sequence	-3.99	6.10E-04
	BB830387	BB830387, EST	-2.46	6.60E-04
	BB341238	BB341238, EST	-5.42	9.10E-04
2210010C04Rik	AK008695	RIKEN cDNA 2210010C04 gene	-5.22	9.50E-04
Eps15l1	NM_001122832	epidermal growth factor receptor pathway substrate 15-like 1	-4.34	1.12E-03
				1.17E-03
Zfp287	NM_133208	Zinc finger protein 287	-5.57	

Gene Symbol	NCBI Accession	Description	Fold Change *Mecp2*-null vs. WT	*P* Value
6430537H07Rik	NM_178689	RIKEN cDNA 6430537H07 gene	-3.00	1.21E-03
Nfatc2	NM_001037177	nuclear factor of activated T-cells, cytoplasmic, calcineurin-dependent 2	-4.70	1.36E-03
Ints7	NM_178632	integrator complex subunit 7	-3.21	1.49E-03
Sidt1	NM_198034	SID1 transmembrane family, member 1	-5.49	1.52E-03
	C76313	C76313, EST	-3.58	1.52E-03
	AK044288	AK044288, EST	-3.45	1.89E-03
Tgm2	NM_009373	ransglutaminase 2, C polypeptide	-7.72	1.93E-03
	AA241963	AA241963, EST	-2.57	2.17E-03
Lmcd1	NM_144799	LIM and cysteine-rich domains 1	-1.90	2.26E-03
Thap7	NM_026909	THAP domain containing 7	-1.72	2.30E-03
	AW124522	AW124522, EST	-4.60	2.41E-03
Gpr150	NM_175495	G protein-coupled receptor 150	-5.80	2.46E-03
Wdr92	NM_178909	WD repeat domain 92	-2.08	2.49E-03
Btbd11	NM_001017525	BTB (POZ) domain containing 11	-2.35	2.57E-03
Gja1	NM_010288	gap junction protein, alpha 1	-2.64	2.65E-03
Ccnd2	NM_009829	cyclin D2	-3.55	2.72E-03
5330425B07Rik	AK019908	RIKEN cDNA 5330425B07 gene	-4.24	2.73E-03
Sumf2	NM_026445	sulfatase modifying factor 2	-6.44	2.92E-03
Il13ra1	NM_133990	interleukin 13 receptor, alpha 1	-4.77	2.99E-03
	BB810913	BB810913, EST	-5.25	3.21E-03
E130317F20Rik	AK148204	RIKEN cDNA E130317F20 gene	-6.10	3.28E-03
Snrnp70	NM_009224	small nuclear ribonucleoprotein 70 (U1)	-4.92	3.31E-03
Traf4	NM_009423	Tnf receptor associated factor 4	-4.22	3.32E-03
Gsn	NM_146120	gelsolin	-1.96	3.33E-03
Prl7a1	NM_008930	prolactin family 7, subfamily a, member 1	-4.06	3.38E-03
Plp1	NM_011123	proteolipid protein (myelin) 1	-2.33	3.39E-03
	T25656	T25656, EST	-4.30	3.42E-03
Plp1	NM_011123	proteolipid protein (myelin) 1	-5.19	3.46E-03

Gene Symbol	NCBI Accession	Description	Fold Change *Mecp2*-null vs. WT	*P* Value
Ccnf	NM_007634	cyclin F	-2.32	3.59E-03
A330068G13Rik	XR_002354	RIKEN cDNA A330068G13 gene	-5.53	3.89E-03
Lcp2	NM_010696	lymphocyte cytosolic protein 2	-4.28	3.92E-03
Fignl1	NM_021891	fidgetin-like 1	-6.56	4.03E-03
Mycn	NM_008709	v-myc myelocytomatosis viral related oncogene	-3.46	4.24E-03
Zxdc	NM_030260	ZXD family zinc finger C	-2.00	4.42E-03
2610002M06Rik	NM_025921	RIKEN cDNA 2610002M06 gene	-1.61	4.64E-03
Bcl7c	NM_009746	B-cell CLL/lymphoma 7C	-2.72	4.75E-03
	AK079106	AK079106, EST	-9.10	4.77E-03
Cdadc1	NM_027986	cytidine and dCMP deaminase domain containing 1	-2.45	4.77E-03
Higd1c	NM_001002900	HIG1 domain family, member 1C	-2.16	4.90E-03
Mecp2	NM_001081979	methyl CpG binding protein 2	-6.08	4.97E-03

Supplementary Table 5: Functional categorization of dysregulated genes in *Mecp2*-null P14 CPN (fold change > 1.5 or < -1.5, P < 0.005)

Dendritic, cytoskeletal, and synaptic formation, maturation, and maintenance

Gene Symbol	NCBI Accession	Description	Fold Change Mecp2-null vs. WT	P Value
Mecp2	BB130002	methyl CpG binding protein 2	-83.78	0.00E+00
Mecp2	BC027153	methyl CpG binding protein 2	-12.71	1.28E-16
Kif1b	BE199508	Kif1b kinesin family member 1B	6.25	2.91E-07
Pde4b	BG793493	phosphodiesterase 4B, cAMP specific	-4.77	2.00E-05
Gsn	NM_010354	gelsolin	-3.65	2.50E-04
Pcdh17	NM_001013753	protocadherin 17	1.76	4.60E-04
Mcf2	NM_133197	mcf.2 transforming sequence	-3.99	6.10E-04
Naaladl2	XM_91083	N-acetylated alpha-linked acidic dipeptidase-like 2	3.83	1.29E-03
Unc5b	NM_029770	Unc5b unc-5 homolog B (C. elegans)	4.80	1.59E-03
Tmem2	NM_001033759	transmembrane protein 2	1.79	1.61E-03
Camk2d	NM_001025438	calcium/calmodulin-dependent protein kinase II, delta	2.00	2.40E-03
Gsn	NM_146120	gelsolin	-1.96	3.33E-03
Plp1	NM_011123	proteolipid protein (myelin) 1	-2.33	3.39E-03
Plp1	NM_011123	proteolipid protein (myelin) 1	-5.19	3.46E-03
Grip1	AK086934	Glutamate receptor interacting protein 1	4.15	4.13E-03
Adora2a	NM_009630	adenosine A2a receptor	2.18	4.82E-03
Mecp2	NM_001081979	methyl CpG binding protein 2	-6.08	4.97E-03

Standardly viewed as immune system-related

Gene Symbol	NCBI Accession	Description	Fold Change Mecp2-null vs. WT	P Value
Cd99l2	BB334959	CD99 antigen-like 2	-3.43	1.36E-06
Irak1	AV091354	interleukin-1 receptor-associated kinase 1	2.80	2.09E-06
Cd99l2	BB334959	CD99 antigen-like 2	-5.79	4.33E-06
Igbp1b	AK006203	immunoglobulin (CD79A) binding protein 1b	-5.67	2.10E-04
Igh-VJ558	AK007826	immunoglobulin heavy chain (J558 family)	4.50	2.30E-04

Klra12	AF204265	killer cell lectin-like receptor subfamily A, member 12	3.09	7.90E-04
Il18bp	NM_010531	Il18bp interleukin 18 binding protein	4.04	1.16E-03
Zfp287	NM_133208	Zinc finger protein 287	-5.57	1.17E-03
Irak1	NM_008363	interleukin-1 receptor-associated kinase 1	2.79	1.24E-03
Irak1	NM_008363	interleukin-1 receptor-associated kinase 1	3.17	2.09E-03
Il13ra1	NM_133990	interleukin 13 receptor, alpha 1	-4.77	2.99E-03
Lcp2	NM_010696	lymphocyte cytosolic protein 2	-4.28	3.92E-03
Zxdc	NM_030260	ZXD family zinc finger C	-2.00	4.42E-03

Metabolism, endocrine

Gene Symbol	NCBI Accession	Description	Fold Change Mecp2-null vs. WT	P Value
Cpm	BB549552	carboxypeptidase M	7.83	3.00E-05
Plekha1	BB475301	Pleckstrin homology domain containing, family A (phosphoinositide binding specific) member 1	5.28	2.00E-05
Lpin2	NM_022882	Lipin 2	4.01	3.49E-03
Casr	NM_013803	calcium-sensing receptor	3.95	4.49E-03
Dep1	BB626684	diabetic embryopathy 1	3.71	9.80E-04
V1rd1	NM_030742	vomeronasal 1 receptor, D1	3.02	9.90E-04
6430537H07Rik	NM_178689	RIKEN cDNA 6430537H07 gene	-3.00	1.21E-03

Reproduction

Gene Symbol	NCBI Accession	Description	Fold Change Mecp2-null over WT	P Value
Cst12	AF440737	cystatin 12	3.97	6.00E-05
Mei1	XM_901649	Mei1 meiosis defective 1	4.08	1.97E-03
Obox5	NM_145709	oocyte specific homeobox 5	2.71	4.04E-03
Ovgp1	NM_007696	oviductal glycoprotein 1	2.36	4.47E-03
Cdadc1	NM_027986	cytidine and dCMP deaminase domain containing 1	-2.45	4.77E-03

Transcription, RNA processing, DNA modification

Gene Symbol	NCBI Accession	Description	Fold Change Mecp2-null vs. WT	P Value
Utp23	AK005831	rRNA-processing protein UTP23 homolog	5.69	4.50E-04
Nfatc1	BB356861	nuclear factor of activated T-cells, cytoplasmic, calcineurin-dependent 1	-4.60	4.60E-04
Nfatc2	NM_001037177	nuclear factor of activated T-cells, cytoplasmic, calcineurin-dependent 2	-4.70	1.36E-03
Ints7	NM_178632	integrator complex subunit 7	-3.21	1.49E-03
Ttll10	NM_029264	tubulin tyrosine ligase-like family, member 10	2.44	1.70E-03
Lmcd1	NM_144799	LIM and cysteine-rich domains 1	-1.90	2.26E-03
Thap7	NM_026909	THAP domain containing 7	-1.72	2.30E-03
Wdr92	NM_178909	WD repeat domain 92	-2.08	2.49E-03
Snrnp70	NM_009224	small nuclear ribonucleoprotein 70 (U1)	-4.92	3.31E-03
Tcfe2a	NM_011548	transcription factor E2a	5.06	4.92E-03

Miscellaneous

Gene Symbol	NCBI Accession	Description	Fold Change Mecp2-null vs. WT	P Value
Rab39b	AK020665	RAB39B, member RAS oncogene family	3.90	4.53E-08
Nhsl2	XM_00147828	NHS-like 2	1.58	1.78E-07
Ankrd17	BG065567	Ankyrin repeat domain 17	-3.66	6.39E-06
Fbxw10	NM_001033669	F-box and WD-40 domain protein 10	1.78	2.00E-05
A630023P12Rik	AI451630	RIKEN cDNA A630023P12 gene	-5.26	7.00E-05
Usp6nl	NM_001080548	USP6 N-terminal like	1.51	1.20E-04
Mycn	NM_008709	v-myc myelocytomatosis viral related oncogene	-1.93	1.40E-04
	BB102018	BB102018, EST	2.25	1.70E-04
Ccdc126	NM_175098	coiled-coil domain containing 126	-1.90	2.40E-04
5830434F19Rik	AK017969	RIKEN cDNA 5830434F19 gene	5.32	2.90E-04
	AW912607	uf54a03.x1, EST	-3.82	3.10E-04
	BQ031094	UI-1-CF0-alz-c-03-0-UI.s1, EST	-2.14	4.50E-04
Tas2r119	NM_020503	taste receptor, type 2, member 119	4.71	4.70E-04
D3Ertd300e	NM_019995	DNA segment, Chr 3, ERATO Doi 300, expressed	-6.54	4.70E-04
C330006K01Rik	AV263612	RIKEN cDNA C330006K01 gene	-4.92	4.80E-04

Hsd17b13	AV050442	hydroxysteroid (17-beta) dehydrogenase 13	-4.13	5.10E-04
Thumpd2	AK012806	THUMP domain containing 2	-4.11	5.80E-04
	BB830387	BB830387, EST	-2.46	6.60E-04
1700082M22Rik	NM_028564	RIKEN cDNA 1700082M22 gene	3.98	6.70E-04
Gpatch8	BC019948	G patch domain containing 8	2.03	6.70E-04
Ptprk	BB667296	protein tyrosine phosphatase, receptor type, K	6.10	8.00E-04
	BB341238	BB341238, EST	-5.42	9.10E-04
2300010F08Rik	AK009054	RIKEN cDNA 2300010F08 gene	4.12	9.30E-04
2210010C04Rik	AK008695	RIKEN cDNA 2210010C04 gene	-5.22	9.50E-04
Eps15l1	NM_001122832	epidermal growth factor receptor pathway substrate 15-like 1	-4.34	1.12E-03
	BM249512	BM249512, EST	3.98	1.37E-03
	C76313	C76313, EST	-3.58	1.52E-03
Sidt1	NM_198034	SID1 transmembrane family, member 1	-5.49	1.52E-03
Gpr107	NM_178760	G protein-coupled receptor 107	1.66	1.63E-03
	AK044288	AK044288, EST	-3.45	1.89E-03
Tgm2	NM_009373	ransglutaminase 2, C polypeptide	-7.72	1.93E-03
Atp2c1	NM_175025	ATPase, Ca++-sequestering	2.85	1.96E-03
A630038E17Rik	BC038285	RIKEN cDNA A630038E17 gene	3.91	2.03E-03
	AA241963	AA241963, EST	-2.57	2.17E-03
LOC552912	XR_005134	hypothetical LOC552912	1.54	2.36E-03
	AW124522	AW124522, EST	-4.60	2.41E-03
Gpr150	NM_175495	G protein-coupled receptor 150	-5.80	2.46E-03
Btbd11	NM_001017525	BTB (POZ) domain containing 11	-2.35	2.57E-03
Wdfy3	XM_152907	WD repeat and FYVE domain containing 3	2.05	2.60E-03
	AK045858	AK045858, EST	2.53	2.64E-03
Gja1	NM_010288	gap junction protein, alpha 1	-2.64	2.65E-03
Ccnd2	NM_009829	cyclin D2	-3.55	2.72E-03
5330425B07Rik	AK019908	RIKEN cDNA 5330425B07 gene	-4.24	2.73E-03
Klk5	NM_008456	Klk1b5 kallikrein 1-related peptidase b5	4.44	2.86E-03

	BE995622	UI-M-CG0p-bia-e-07-0-UI.s1, EST	3.47	2.89E-03
Sumf2	NM_026445	sulfatase modifying factor 2	-6.44	2.92E-03
	BB810913	BB810913, EST	-5.25	3.21E-03
E130317F20Rik	AK148204	RIKEN cDNA E130317F20 gene	-6.10	3.28E-03
Traf4	NM_009423	Tnf receptor associated factor 4	-4.22	3.32E-03
Prl7a1	NM_008930	prolactin family 7, subfamily a, member 1	-4.06	3.38E-03
4933439C20Rik	NR_003518	RIKEN cDNA 4933439C20 gene	1.64	3.39E-03
	T25656	T25656, EST	-4.30	3.42E-03
Ccnf	NM_007634	cyclin F	-2.32	3.59E-03
4933402E13Rik	XM_889482	RIKEN cDNA 4933402E13 gene	2.89	3.74E-03
5033405D04Rik	AK019887	RIKEN cDNA 5033405D04 gene	2.38	3.85E-03
A330068G13Rik	XR_002354	RIKEN cDNA A330068G13 gene	-5.53	3.89E-03
Neurl3	NM_153408	Neurl3 neuralized homolog 3 homolog (Drosophila)	4.92	3.94E-03
Fignl1	NM_021891	fidgetin-like 1	-6.56	4.03E-03
Mycn	NM_008709	v-myc myelocytomatosis viral related oncogene	-3.46	4.24E-03
D18Ertd653e	NM_172631	DNA segment, Chr 18, ERATO Doi 653, expressed	3.56	4.31E-03
2610002M06Rik	NM_025921	RIKEN cDNA 2610002M06 gene	-1.61	4.64E-03
Rab34	NM_033475	RAB34, member of RAS oncogene family	1.84	4.68E-03
	AK084017	AK084017, EST	1.60	4.69E-03
Bcl7c	NM_009746	B-cell CLL/lymphoma 7C	-2.72	4.75E-03
	AK079106	AK079106, EST	-9.10	4.77E-03
Higd1c	NM_001002900	HIG1 domain family, member 1C	-2.16	4.90E-03

Supplementary Methods:

Bisulfite genomic sequencing

Genomic DNA was extracted from three independent cortices of wild-type P14 male C57BL/6 mice using the DNeasy Blood & Tissue kit according to the manufacturer's instructions (Qiagen, Valencia, CA). 500 ng of genomic DNA was used for bisulfite treatment using the EZ DNA Methylation-Gold kit (Zymo Research, Orange, CA). Bisulfite-modified DNA was amplified by PCR using the following six primer pairs covering 79 CpGs on the promoter region of *Irak1*.

Pair 1:	Forward	5'- GAG GAT AAT GGT ATG TTA TAA -3'
	Reverse	5'- TTA ACC ATA TAC TAA ACT TCC -3'
Pair 2:	Forward	5'- GTT AGT TAT GGA TTT TGT GTA -3'
	Reverse	5'- CTA AAC CCA ACT CCT CTC TTA -3'
Pair 3:	Forward	5'- GTG TAT TTA TAT GAA GAG GAT -3'
	Reverse	5'- AAT ATA TAC CAT ACT ACT ACT-3'
Pair 4:	Forward	5'- GAT TGA TTA ATA ATT TGG TTG -3'
	Reverse	5'- AAT CTATAA TCC TAC ACAAAC -3'
Pair 5:	Forward	5'- TTA GAT GTT TGT GTA GGA TTA TAG TTT -3'
	Reverse	5'- ACA CAT AAC CCA AAA TAA CAC CT -3'
Pair 6:	Forward	5'- AGG TGT TAT TTT GGG TTA TGT -3'
	Reverse	5'- TCA CAA ACT ATA ATA ATA TCC -3'

The PCR products were subcloned into the pCRII-TOPO vector (Invitrogen), and 10 clones were sequenced for each PCR product (for a total of 30 clones from 3 independent P14 cortical samples).

Chromatin immunoprecipitation (ChIP)

Cortices were dissected from three P14 mice for each ChIP experiment, and dissociated as described above. ChIP was performed three times using the EZ ChIP kit (Millipore, Billerica, MA). Approximately six million dissociated cortical cells were fixed in 1% paraformaldehyde at room temperature for 10 min, and unreacted paraformaldehyde was then quenched by adding glycine solution (final 0.125 M). After washing the cross-linked dissociated cells with ice-cold PBS twice, the cells were lysed in 600 μl SDS lysis buffer [1% SDS, 10 mM EDTA, 50 mM Tris-HCl (pH 8.1), protease inhibitor cocktail]. The lysate was sonicated on ice with a Branson Sonifier 450 (Branson, Danbury, CT) using a double stepped microtip (tip diameter 3 mm) for 10 sec, 12 times, at power level 1, to produce fragments with an average length of ~100 to 1,000 bp. 100 μl of sonicated chromatin was added to 900 μl of ChIP dilution buffer (Millipore). The sample was precleared with Protein G Agarose (Millipore) for one hour at 4 °C, and 1% of the sample was set aside as an input sample. The precleared lysate was incubated with either 5 μg of rabbit polyclonal anti-MeCP2 antibody (Millipore) or 5 μg of rabbit IgG (Sigma-Aldrich, St. Louis, MO) overnight at 4°C. Protein-DNA-antibody complexes were precipitated with Protein G Agarose, and were washed sequentially with low salt buffer, high salt buffer, and TE buffer (Millipore). The precipitated protein-DNA-antibody complexes were eluted from Protein G Agarose with 1% SDS and 0.1 M $NaHCO_3$, and then incubated at 65 °C for 5 hours in 200 mM NaCl to reverse formaldehyde cross-links. Following RNase A and proteinase K treatment, the sample was purified by phenol/chloroform extraction and ethanol precipitation. The IP samples were subjected to PCR amplification using the following primer pairs that cover the 5 highly methylated CpGs on the *Irak1* promoter:

Pair 1 (for -2107 bp CpG)

Forward 5'- ATT GTG AGC ATA CCT AGA AGC -3'

Reverse 5’- CCA AGA GGT TAA CCATAT GCT -3’

Pair 2 (for -1492 bp CpG)

Forward 5’- TCT GAA GAC ATA CTA TAA GTT -3’

Reverse 5’- TGA ACA CAC CTT GAG CTT GCT -3’

Pair 3 (for -779, -547, and -426 bp CpGs)

Forward 5’- CAG AGA GCA TAA TGA GAA AGT -3’

Reverse 5’- TTG TGT GCT AGA TCT CCA TGC -3’
